# ADHD and Suicidality in Adolescents: A Systematic Review of Moderators and Mediators

**DOI:** 10.1007/s10567-025-00531-9

**Published:** 2025-06-19

**Authors:** Yvette Rother, Carissa M. Orlando, Peter Warren, Kate Flory

**Affiliations:** Department of Psychology, University of South Carolina, Barnwell College, 1512 Pendleton Street, Columbia, SC 29208 USA

**Keywords:** ADHD, Suicidality, Adolescence, Risk factors, Protective factors

## Abstract

In the United States, suicide is the second leading cause of death for those aged 10–14 and the third for those aged 15–24. Adolescents with attention-deficit/hyperactivity disorder (ADHD) are at an increased risk for suicide compared to adolescents without ADHD. However, the strength of the relation between ADHD and suicide and the mechanisms at play in this association are unclear and need further investigation. This systematic review examined the literature to determine whether there is a direct association between ADHD and suicidality in adolescents, and which factors moderate or mediate this relation. A systematic literature search, adhering to the Preferred Reporting Items for Systematic Reviews and Meta-Analyses (PRISMA) guidelines, was carried out on December 20th, 2024. Relevant publications were identified via electronic searches of PsycInfo and Web of Science databases. Studies were included if they assessed ADHD, suicidality, and at least one additional construct analyzed as a moderator or mediator. Studies had to include participants with a mean age between 10 and 19 years (i.e., an adolescent sample). The literature search yielded 405 articles, with 28 included in the final study selection. ADHD emerged as an independent risk factor for suicide among adolescents. Various constructs, including psychological, demographic, family, peer and social, subjective well-being, and trauma and adversity factors, served as moderators or mediators to varying degrees. Our review can inform adolescent suicide prevention efforts by highlighting the importance of addressing specific risk factors. Further research is needed to examine understudied factors, with emphasis on strengths-based factors to improve well-being.

**Supplementary Information:**

The online version contains supplementary material available at 10.1007/s10567-025-00531-9.

## ADHD and Suicidality in Adolescents: A Systematic Review of Moderators and Mediators

Attention-deficit/hyperactivity disorder (ADHD), recognized as one of the most prevalent childhood neurodevelopmental disorders (Wigal et al., 2013), affects approximately 9.8% of children and adolescents aged 3–17 (Bitsko et al., 2022). The disorder is marked by symptoms such as inattention, hyperactivity, and/or impulsivity, resulting in functional impairment in at least two settings, with symptoms emerging before the age of 12 years (American Psychiatric Association [APA], 2022). Several negative outcomes are associated with ADHD in a youth population. For example, research indicates that, compared to their peers without ADHD, youth exhibiting heightened ADHD symptoms face a greater risk of academic underachievement, including lower GPAs, and an increased likelihood of grade repetition (Loe et al., 2007). Elevated levels of ADHD symptoms during childhood have also been linked to subsequent unhealthy behaviors and lifestyle choices (Holton & Nigg, 2020), including an increased likelihood of substance use during adolescence and adulthood (Whalen et al., 2002). Moreover, Reale and colleagues (2017) found that children and adolescents diagnosed with ADHD were notably more prone to psychiatric comorbidities, with 66% exhibiting at least one additional psychiatric disorder, including learning disorders (56%), sleep disorders (23%), oppositional defiant disorder (20%), and anxiety disorders (12%).

Additionally, social difficulties (e.g., poorer communication skills) and emotional challenges (e.g., difficulty regulating emotions, heightened expressions of anger and aggression) associated with ADHD significantly impact the well-being of both youth and their families (Wehmeier et al., 2010). Adolescents with ADHD symptoms demonstrated lower scores across all dimensions of health-related quality of life (in physical, emotional, self-esteem, family, friends/peers, and school/education domains) compared to those without notable ADHD symptoms (Krauss & Schellenberg, 2022). Emerging evidence in the literature suggests that an increase in ADHD symptoms and related negative outcomes are associated with lower life satisfaction (Gudjonsson et al., 2009; Nadeau et al., 2015). Low life satisfaction has a long-term effect on the risk of suicide (Koivumaa-Honkanen et al., 2001). Additionally, the aforementioned negative outcomes (e.g., academic problems, psychiatric comorbidities, and social difficulties) have been linked to an increased risk of suicidality (Arun et al., 2017; Nock et al., 2010). Therefore, considering the numerous challenges and adverse outcomes people with ADHD may experience, it is important to explore the relation between suicidality and ADHD to better understand the factors contributing to risk and to develop targeted interventions. The current systematic review adds to existing literature by further clarifying the ADHD-suicidality relation in adolescents and examining the role of potential moderators and mediators, contributing to a more comprehensive understanding of factors that may influence this association (e.g., strengthen or weaken the relationship, shape pathways).

### Suicide in Adolescence

Adolescence is a pivotal developmental stage with distinctive biological processes, environmental and social influences, and social-emotional changes that can impact well-being and risk-taking behaviors (Institute of Medicine [US] & National Research Council [US] Committee on the Science of Adolescence, 2011). For example, adolescents are particularly vulnerable due to ongoing neurodevelopmental changes affecting areas crucial for risk-taking behaviors, particularly brain areas related to reward, inhibitory control, and emotional processing (Spear, 2013). Moreover, early adolescence is a critical time when youth face numerous life changes, including puberty and transitions at home and school, which challenge their ability to cope and adapt, revealing varying patterns of competence or distress (Roeser et al., 2002). Further, environmental stressors like peer relationships, academic demands, family dynamics, and increased autonomy present unique challenges in adolescence (Brown & Larson, 2009; Jaworska & MacQueen, 2015; Roeser et al., 2002). Negative experiences in these areas were found to have a positive association with suicidality (Fotti et al., 2006).

In the United States, suicide ranks as the second leading cause of death among individuals aged 10–14 and the third leading cause of death among individuals aged 15–24 (Centers for Disease Control and Prevention [CDC], 2021). Suicide profoundly affects families and communities, leaving lasting emotional and social impacts (Cerel et al., 2008). Developmental studies show that the highest occurrence of suicidal ideation typically occurs during mid-adolescence (Lewinsohn et al., 1996; Rueter & Kwon, 2005). The current study examines “suicidality” as an outcome, encompassing diverse components of suicide, including suicidal ideation, suicidal plans, and suicide attempts. Although no universal definition exists, suicidal ideation is a broad term that refers to thoughts, fantasies, or preoccupations about death and taking one’s own life (Harmer et al., 2024). Suicidal thoughts can vary in intensity from fleeting to persistent desires or plans, with over a third of those with ideation progressing to suicide attempts (Nock et al., 2013). A suicide attempt is a deliberate, self-inflicted action intended to end one’s life, but it does not necessarily result in death (Crosby et al., 2011). The transition from ideation to attempts involves complex factors, including the individual’s capacity to overcome the fear of death (Klonsky et al., 2016). Using data from 2021, the reported prevalence of attempted suicide was 13.3% in female adolescents and 6.6% in male adolescents (Gaylor et al., 2023). A history of suicide attempt strongly predicts death by suicide (Bostwick et al., 2016).

### ADHD and Suicidality

Although suicidality is highly prevalent, research on identifying specific population subgroups at heightened risk for suicide remains relatively limited when contrasted with studies examining suicide risk at the broader population level (Evans et al., 2017). Among vulnerable subgroups, adolescents with ADHD emerge as particularly noteworthy, as they may be at heightened risk for suicidality compared to the general population due to increased impulsivity, executive dysfunction, emotion dysregulation, and ADHD-related problems (Bredemeier & Miller, 2015; Groves et al., 2022; Turton et al., 2021; Whiteside & Lynam, 2001). Lower educational attainment, childhood maltreatment, and delinquency have also been found to contribute to the risk of suicidality (Impey & Heun, 2012; Todzia-Kornas et al., 2024) in adolescents with elevated ADHD symptoms.

Indeed, the association between ADHD and suicidality has garnered much attention within the research literature, leading to the publication of 11 review and summary papers on the topic (Austgulen et al., 2023; Balazs & Kereszteny, 2017; Furczyk & Thome, 2014; Garas & Balazs, 2020; Giupponi et al., 2018; Hinshaw et al., 2022; Impey & Heun, 2012; James et al., 2004; Nigg, 2013; Septier et al., 2019; Todzia-Kornas et al., 2024). While 10 of these papers included articles on adolescents, none solely focused on this age group. Furthermore, while all reviews indicate an overall significant positive association between ADHD and suicidality, only two have established this link across multiple age groups, including adolescents (Austgulen et al., 2023; Septier et al., 2019). The remaining studies did not assess the magnitude of these associations and noted that various moderators and mediators may explain significant findings. This suggests that, while there is a general consensus on the association and some evidence for ADHD independently increasing the risk of suicidality, the specific mechanisms and strength of this relation warrant further investigation. Therefore, it is important to examine factors contributing to suicidality in an adolescent population, particularly those with ADHD who are at an increased risk (Austgulen et al., 2023).

To date, the majority of review articles addressing ADHD and suicidality have not intentionally examined the influence of mediating or moderating variables in adolescents with ADHD, which could result in a biased assessment of the relation between ADHD and a risk of suicidal spectrum behaviors (SSBs), such as suicidal ideation, attempts, plans, and completed suicides (Septier et al., 2019). Although some review articles have identified several contributing variables in their included studies, particularly noting sex/gender differences and psychiatric comorbidities (Balazs & Kereszteny, 2017; Hinshaw et al., 2022; Impey & Heun, 2012; James et al., 2004; Nigg, 2013; Todzia-Kornas et al., 2024), the possibility remains that important moderators and mediators have been overlooked.

Similar to the current review, Austgulen and colleagues (2023) conducted a systematic review in March of 2022 to investigate factors that may increase the risk of SSBs in adults and adolescents with ADHD. Their final study selection included 40 studies. The factors associated with an increased likelihood of SSBs included severity and persistence of ADHD symptoms, female gender, family history of ADHD, childhood and parental influences, and social functioning. However, unlike the current review, Austgulen and colleagues’ review only included studies examining mediating factors, not also moderating factors. Additionally, the included studies required samples with an ADHD diagnosis specified by the Diagnostic and Statistical Manual of Mental Disorders (DSM) or International Classification of Diseases (ICD) criteria and a mean age above 16 at either baseline or assessment for SSBs. However, subthreshold disorders (i.e., those who do not fulfill all the criteria) have been found to also increase suicidal risk in adolescents, highlighting the importance of also examining the association of suicidality and ADHD-related symptomology (Balazs et al., 2014). Additionally, several differences in search terms (self-harm, self-injury, self-mutilation, suicide, self-poisoning, adults, adolescents, attention-deficit hyperactivity disorder/ADHD), electronic databases (Ovid MEDLINE, Web of Science), and inclusion and exclusion criteria between the Austgulen and colleagues’ study and the current study resulted in an overlap of only six studies included in the two reviews. Therefore, the current study is distinct and expands on the prior findings.

Examining moderating and mediating factors in the relation between ADHD and suicidality is important because it can help identify specific variables that influence the risk of suicidality among adolescents with ADHD. There are a number of demographic, psychological, and social factors that could influence or explain this relation. Understanding the nuanced interplay between these factors and suicidality can lead to the development of more tailored and effective intervention strategies, ultimately improving the overall well-being and quality of life for adolescents with ADHD who may be at risk of suicidality.

### Current Review

As previously noted, existing reviews have extensively explored the association between ADHD and suicidality. However, when comparing the studies included in these prior reviews with those in the current review, there is minimal overlap, with only zero to eight studies shared between them. This limited overlap suggests that the current review incorporates new research or presents a different focus compared to previous reviews, highlighting a potential gap in the existing literature. Specifically, this gap may reflect a lack of comprehensive synthesis on mediating or moderating factors in younger populations or those who exhibit ADHD symptoms, as most prior reviews have focused on the lifespan or adult samples, those with ADHD diagnoses, or did not intentionally synthesize factors that moderate or mediate the ADHD-suicidality linkage. As a result, critical insights into developmental differences, risk and protective factors specific to adolescence, or the role of subclinical ADHD symptoms in shaping suicidality risk may be overlooked, limiting the ability to inform targeted interventions. To date, we are only aware of one systematic review that intentionally examined the literature to determine mediating factors among late adolescent and adult samples with an ADHD diagnosis and SBBs (Austgulen et al., 2023).

Considering these aforementioned gaps, the current systematic review aimed to provide an extension of previous research by synthesizing literature to answer the following questions: (1) Is there a direct association between ADHD and suicidality in adolescents? and (2) What factors moderate or mediate this association? This review can provide clinicians with insights into identifying risk factors to help prevent crises, develop more effective treatment approaches, and guide targeted interventions for adolescents with ADHD. Additionally, a deeper understanding of moderating and mediating factors in the ADHD-suicidality relation contributes to the literature and can inform future research directions.

## Methods

The protocol for this review was not pre-registered in a public database. The review process followed a structured methodology, which included predefined inclusion and exclusion criteria, a systematic search strategy, and a standardized study selection procedure. Study selection and data extraction were conducted by a single reviewer using a two-stage process: initial title and abstract screening, followed by full-text review of studies that met preliminary criteria. Eligibility criteria were applied at both stages, based on the predefined inclusion and exclusion criteria described below. Both study selection and data extraction were conducted manually using a standardized Excel spreadsheet. Decisions and reasoning regarding inclusion and exclusion were recorded in the spreadsheet. The extracted data from full-text reviews included study authors, publication year, country of origin, sample size, population characteristics (e.g., age), variables of interest (e.g., ADHD, suicidality), study design, analytical approach (e.g., moderation, mediation), key findings, and whether the study was empirical and peer-reviewed. Any missing or unclear information was noted.

### Inclusion and Exclusion Criteria

To be included in this review, studies had to meet several criteria. First, the study was required to measure ADHD, suicidality, and at least one additional construct that served as a moderator or mediator in the analyses. All three components must have been present for inclusion. The measurement of ADHD could have been approached categorically (by determining if an ADHD diagnosis was present or absent) or continuously (by examining the number of ADHD symptoms reported) using various techniques such as diagnostic interviews and standardized rating scales. Additionally, suicidality could have been captured through constructs such as suicidal ideation, plans, or attempts. Second, the target population for this review was adolescents, defined by the World Health Organization as individuals aged 10 to 19 years. Consequently, eligible studies needed to report a mean sample age within this range, with longitudinal studies requiring that participants’ mean age fall within this range during the assessment of suicidality. Finally, the study had to be published in English and be both peer-reviewed and empirical in nature.

Studies were excluded if they did not measure ADHD, suicidality, and an additional construct analyzed as a moderator or mediator. If the mean age of participants was outside the 10 to 19 age range, or studies were published in languages other than English, were not peer-reviewed, or lacked an empirical basis, they were excluded. Studies focused on the effectiveness and safety of pharmacological ADHD treatments and suicidality were not included either because this area has already been extensively covered in recent literature reviews (Chen et al., 2014; Man et al., 2017). Studies that considered ADHD medication status as one of the variables were included, provided they were not clinical trials where medication status served as the independent variable.

### Search Strategy

The review was conducted following the Preferred Reporting Items for Systematic Review and Meta-Analysis (PRISMA) guidelines (Page et al., 2021). Two databases were searched (PsycInfo, Web of Science) on December 20th, 2024 using the following search terms: “ADHD” OR “attention-deficit hyperactivity disorder” AND “adoles*” OR “teen*” OR “youth*” OR “child*” AND “suicide*” AND “moderate*” OR “mediate*” OR “risk factor” OR “mechan*” OR “predict*” OR “pathway” OR “interact*” OR “protective factor” OR “protective.”

### Study Selection

**Fig. 1:**
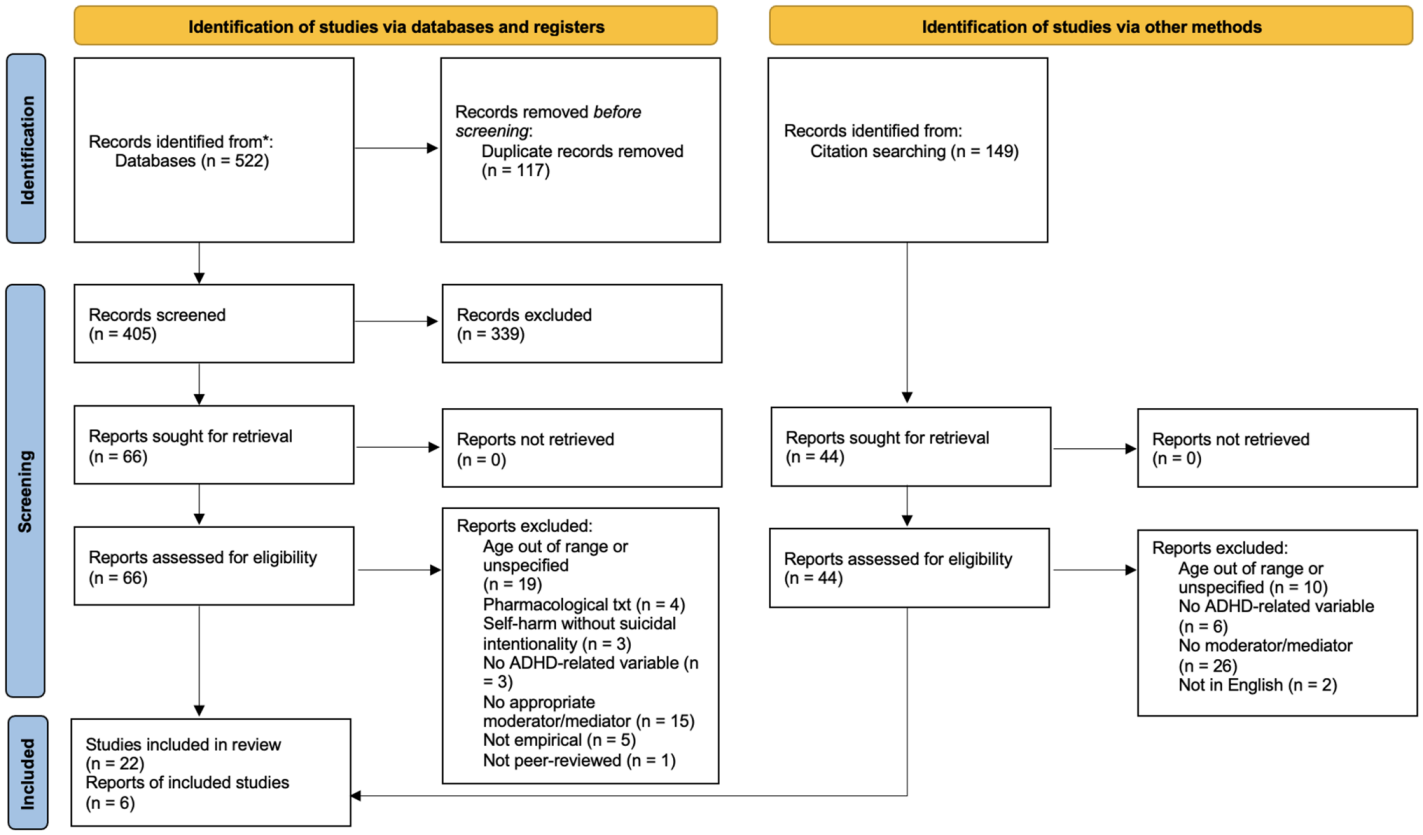
Prisma Flow Diagram

Article identification, screening, and inclusion information is depicted in Fig. 1. The search yielded 177 articles from PsycInfo and 345 articles from Web of Science. After removing 117 duplicates, the total number of unique articles that remained was 405. Abstracts were reviewed to identify potentially eligible articles resulting in 66 full-text articles then reviewed for eligibility criteria. Additionally, references from previous review articles (Austgulen et al., 2023; Balazs & Kereszteny, 2017; Giupponi et al., 2018; Impey & Heun, 2012; Septier et al., 2019) were included in the search to identify additional relevant articles to include in the review. The references of eligible articles were also examined to identify additional relevant articles to include in the review that were missed upon the initial search. This process yielded 44 additional full-text articles that were reviewed for eligibility criteria. In total, 28 articles met criteria to be included in the review.

## Results

### Demographic Characteristics

Demographic characteristics can be found in Supplemental Table 1. The included studies focused on adolescents with a mean age between 10 and 19 years. Several studies (*n* = 12) collected longitudinal data; therefore, at least one wave that examined suicidality had to include participants with a mean age within our target range. Nearly all studies reported sex or gender, with about half of the studies having either male-dominated samples or a relatively balanced distribution. The term “sex/gender” is used to include all studies that examined either sex or gender and to reflect the complex interplay of social and biological influences (Coen & Banister, 2012). Six studies exclusively included female participants (Cho et al., 2008; Gordon & Hinshaw, 2017; Guendelman et al., 2016; Hinshaw et al., 2012; Meza et al., 2016; Swanson et al., 2014), and one study focused solely on male participants (Ruchkin et al., 2017). The majority of the studies were conducted in the United States (*n* = 13), with others carried out in Taiwan (*n* = 3), Canada (*n* = 2), Hungary (*n* = 2), South Korea (*n* = 2), Austria (*n* = 1), Australia (*n* = 1), China (*n* = 1), Israel (*n* = 1), Russia (*n* = 1), and Sweden (*n* = 1). Nearly all U.S. studies reported race and/or ethnicity distributions, with the majority of participants being White. Only two international studies reported participant race or ethnicity, such that their samples were 92.5% of Han ethnicity and 7.5% other ethnicities (Zhong et al., 2021), as well as 98% ethnic Slavs (Ruchkin et al., 2017).

**Table 1:** Summary of full-text articles that examined direct effects

Study	Covariates or mediators	Findings
Balazs et al. (2018)	Covariates: Gender, age Mediators: Quality of life	No, the direct effect of hyperactivity/inattention on suicidal risk was not significant (b_3_ = -.477) after considering the mediating effect of quality of life
Chen et al. (2019)	Covariate: Demographic variables (academic year, sex, fathers’ educational attainment, mothers’ educational attainment, parental marital status) Mediators: Anxiety/depression, conduct problems, family function	Yes, ADHD had a significant effect on increasing the likelihood of suicidal ideation, suicide plans, and suicide attempts when controlling for demographic variables (suicide ideation: OR = 3.82, 95% CI [2.73, 5.34]; suicide plan: OR = 4.18, 95% CI [2.57, 6.80]; suicide attempts: OR = 4.45, 95% CI [1.99, 9.93]) Yes, the direct effect of ADHD on the risk of suicidal ideation (*β* = 0.89, *p* < .001) and suicidal plans (*β* = 0.69, *p* < .01) remained statistically significant after considering the mediating effects of both family function and symptoms of anxiety/depression. The direct effect was not significant for suicide attempts due to small sample size (*β* = 0.69, *p* = .07) Yes, the direct effect of ADHD on the risk of suicidal ideation (*β* = 0.99, *p* < .001), suicidal plans (*β* = 0.82, *p* < .01), and suicide attempts (*β* = 0.82, *p* < .05) remained statistically significant after considering the mediating effects of both family function and symptoms of conduct problems
Cho et al. (2008)	Mediator: Depressive symptoms	Yes, the direct effect of conduct problems on suicidal ideation was significant (*β* = 0.13, *p* = 0.000) after considering the mediating effect of depressive symptoms in an all-female sample No, the direct effects of cognitive problems and hyperactivity problems on suicidal ideation were not significant (*β* = 0.01, *p* = 0.808 and *β* = 0.04, *p* = 0.306, respectively) after considering the mediating effect of depressive symptoms in an all-female sample
Chronis-Tuscano et al. (2010)	Covariates: Maternal history of depression, sex	Yes, ADHD had a significant effect on increasing the likelihood of concrete suicidal ideation (χ^2^ = 5.38, *p* = 0.03; hazard ratio, 5.79) and suicide attempts (χ^2^ = 8.26, *p* = 0.005; hazard ratio, 3.60) when controlling for significant effects of sex and maternal history of depression
Katzenmajer-Pump et al. (2022)	Mediators: Depressive symptoms, anxiety symptoms	No, the direct effect of ADHD on suicidal thoughts and planning was not statistically significant after considering the mediating effects of anxiety and depression
Kessler et al. (2014)	Mediators: Temporally secondary psychiatric disorders (mood, anxiety, disruptive behavior, substance abuse)	Yes, the direct effect of ADHD on suicidal ideation and plans was significant in males only (19.3% and 24.2%, respectively) after considering the mediating effects of temporally secondary psychiatric disorders. However, the direct effect was not significant among females
Kim et al. (2015)	Covariates: Age, gender, depressive symptoms	Yes, performance on visual sustained attention tasks (omission errors, commission errors, and reaction time) had a significant effect on suicidal ideation (*p* < 0.001, *p* = 0.019, and *p* = 0.028, respectively) when controlling for age, gender, and depressive symptoms
Lan et al. (2015)	Covariates: Demographic variables (age, sex, SES, level of urbanization), psychiatric comorbidities (anxiety disorder, disruptive behavior disorders, alcohol use disorders, and substance use disorders)	Yes, ADHD had a significant effect on risk of suicide attempts among those with bipolar disorder when controlling for demographic variables and psychiatric comorbidities (hazard ratio: 20.32, 95% CI [8.96, 46.08])
Lin et al. (2024)	Covariates: Sociodemographic variables (Index of Relative Socio-economic disadvantage, maternal age at birth, maternal education) Mediators: Depression, bullying victimization	Yes, ADHD had a significant effect on increasing the likelihood of suicidal thought or attempt when controlling for sociodemographic variables (OR = 11.13, 95% CI [1.92, 64.63], *p* < .01) Yes, the direct effect of ADHD on suicidal thoughts and planning was statistically significant after considering the mediating effects of depression (*β* = 2.45, 95% CI [0.65, 4.25], *p* < .01) and bullying victimization (*β* = 2.48, 95% CI [0.70, 4.26], *p* < .01)
Meza et al. (2016)	Covariates: Sociodemographic variables (household income, age), cognitive variables (child IQ, mother’s education) Mediators: Social preference	Yes, commission errors (response inhibition) had a significant effect on increasing suicide attempts when controlling for sociodemographic and cognitive variables (*β* = .170, *p* < .05, Δ*R*^2^ = .03) in an all-female sample. Results were also significant for suicidal ideation, although the significance was marginal (*β* = .133, *p* = .064, Δ*R*^2^ = .02) Yes, commission errors (response inhibition) had a significant effect on increasing suicidal ideation and suicide attempts after considering the mediating effect of social preference in an all-female sample
Ruchkin et al. (2017)	Covariates: Psychiatric comorbidities (MDD, mania, anxiety disorder, PTSD, CD, alcohol dependence, drug dependence)	Yes, ADHD had a significant effect on increasing the likelihood of suicidal ideation (explained an additional 5.6% of variance) and suicide attempts (explained an additional 7.5% of variance) when controlling for comorbidity in an all-male sample
Swanson et al. (2014)	Mediators: Internalizing symptoms	Yes, ADHD had a significant effect on increasing the likelihood of suicidal ideation after considering the mediating effect of internalizing symptoms (*b* = .28, SE = 13, *t*(195) = 4.12, *p* < .001, R^2^ = .08) in an all-female sample
Vuijk et al. (2019)	Covariates: Age, sex, psychiatric comorbidities	No, ADHD did not have a significant effect on increasing the likelihood of STBs when controlling for sex, age, and comorbid disorders (OR = 1.00, *z* = -0.02, 95% CI [0.67, 1.48],* p* = 0.99)
Zahid et al. (2020)	Covariates: Demographic variables (age, sex, SES, race), psychiatric comorbidities (impulse control disorders, anxiety disorders, psychotic disorders, alcohol abuse, substance abuse)	Yes, ADHD had a significant effect on increasing the likelihood of suicidal behaviors (OR = 1.04, 95% CI [1.01, 1.08]) when controlling for demographic variables and psychiatric comorbidities
Zhong et al. (2021)	Mediators: Depressive symptoms, anxiety symptoms	No, ADHD did not have a significant effect on increasing the likelihood of suicidal ideation after considering the mediating effects of anxiety and depressive symptoms. There were no statistically significant direct paths from inattention (*B* = − 0.024, *β* = 0.046, *p* = 0.592), executive dysfunction (*B* = 0.067, *β* = 0.046, *p* = 0.145), and hyperactivity (*B* = 0.049, *β* = 0.040, *p* = 0.225) to suicidal ideation

### ADHD Characteristics

This systematic review found that ADHD was assessed comprehensively across the literature, as evidenced by the diverse methods employed in the 28 included studies. Of these, 19 studies focused on ADHD diagnoses, 10 examined ADHD symptoms and/or related constructs, and two explored ADHD subtypes. Regarding the reporter, 15 studies incorporated self-report measures or computerized tasks, 13 studies utilized parent-report measures, seven studies relied on teacher-report measures, and one study did not specify. Furthermore, two studies included participants who had previously received an ADHD diagnosis from a clinical provider, which was subsequently confirmed by the study team, and four studies selected participants through a medical record review (e.g., Psychiatric Central Research Register [PCRR]) using codes from the International Classification of Diseases (ICD). Various unique questionnaires, interviews, and tests (*n* = 14) were conducted to measure the ADHD variable, with seven using versions of the Kiddie Schedule for Affective Disorders and Schizophrenia for School-Age Children (K-SADS), five using the Diagnostic Interview Schedule for Children (4th ed.; DISC–IV), four using the Mini International Neuropsychiatric Interview for Children and Adolescents (M.I.N.I. Kid), and three using the Swanson, Nolan, and Pelham, Version IV (SNAP-IV). See Table 1 for additional measures.

### Suicidality Characteristics

Suicidality encompasses a wide range of constructs in the literature, reflecting the complex and multifaceted nature of this phenomenon. Across the 28 included studies, suicidal ideation, suicidal plan/planning, suicide attempts, and suicidal gestures/behaviors (e.g., self-injury alongside suicidal ideation, talking about suicide) were unique constructs of suicidality. Suicide attempts only (*n* = 14), suicidal ideation/thoughts only (*n* = 11), combined suicidal ideation and attempts (*n* = 8), suicidal gestures/behaviors (*n* = 2), suicide plan (*n* = 2), and suicidal thoughts and planning (*n* = 1) were assessed. Most studies relied on self-report measures, though a few also utilized additional parent-report (*n* = 8) or examined medical records (*n* = 4) for ICD codes capturing suicide and self-inflicted injury (e.g., E950–E959). Multiple interviews and questionnaires were utilized (*n* = 18), such as five studies using versions of the KSADS, four studies utilizing the Barkley Suicide Questionnaire, and three studies using the M.I.N.I. Kid.

### Independent Effects of ADHD on Suicidality

The first research question aimed to determine whether a direct association between ADHD and suicidality exists. Of the 28 included studies, 15 assessed the direct effects of ADHD on suicidality while considering mediating effects or controlling for various factors; see Table 1 for more information. Among these 15 studies, 11 found a direct association between ADHD-related constructs and increased risks of suicidality, while 5 did not find significant direct effects. For example, Chen and colleagues (2019) found that children with ADHD are approximately three times more likely to experience suicidal ideation and suicide plans compared to their non-ADHD peers. This heightened risk for suicidality persisted even when controlling for sociodemographic variables such as sex/gender, age, and socioeconomic status, and various parental factors (Chen et al., 2019; Chronis-Tuscano et al., 2010; Kim et al., 2015; Lan et al., 2015; Lin et al., 2024; Meza et al., 2016; Zahid et al., 2020), as well as some psychiatric comorbidities (Kim et al., 2015; Lan et al., 2015; Ruchkin et al., 2017; Zahid et al., 2020). Moreover, the link between ADHD and suicidality remained robust even when accounting for the mediating effects of various psychiatric comorbidities and symptoms (e.g., anxiety/depression, conduct problems, internalizing symptoms), bullying victimization, social preference, and family function (Chen et al., 2019; Cho et al., 2008; Kessler et al., 2014; Lin et al., 2024; Meza et al., 2016; Swanson et al., 2014).

However, three studies that examined the mediating effects of anxiety and/or depression, did not find a significant direct effect of ADHD on suicidality (Cho et al., 2008; Katzenmajer-Pump et al., 2022; Zhong et al., 2021). While an additional study suggested that the effect of ADHD may diminish when controlling for comorbid conditions (Vuijk et al., 2019) and another found no significant direct effects of hyperactivity/inattention on suicidal risk when considering the mediating effect of quality of life (Balazs et al., 2018), overall, the literature supports a direct association between ADHD and increased risks of suicidality.

### Exploring Moderators and Mediators

The second research question aimed to determine what factors moderate or mediate the association between ADHD and suicidality. Refer to Table 2 for a summary of the findings. The identified articles encompassed a range of constructs serving as moderators or mediators, including psychological, demographic, family, peer and social, subjective well-being, and trauma and adversity factors.

**Table 2:** Summary of findings

Broad Category	Subcategory	Broad Cat. Count (Subcat. Count)	Specific Factor (Moderator)	Specific Factor (Mediator)	Study	Main Findings
Psychological	Affective disorders	15 total (Including: 11 Affective disorders; 6 Anxiety disorders; 4 Disruptive behavior disorders; 3 Alcohol use and substance use disorders; 4 ADHD-related factors; 2 Other psychological and behavioral symptoms; 1 Autism spectrum disorder, 1 Posttraumatic stress disorder)	–	Depressive symptoms	Cho et al. (2008)	Depressive symptoms partially mediated the relation between ADHD symptoms (conduct problems) and suicidal ideation in an all-female sample. Depressive symptoms fully mediated the relation between ADHD symptoms (cognitive, hyperactive problems) and suicidal ideation. Collectively, the conduct, cognitive, and hyperactivity problems of the ADHD symptoms and the depressive symptoms accounted for 32.1% of the variance in suicidal ideation
			Mood disorders (interaction term)	–	Kelly et al. (2004)	Mood disorders (major depression, bipolar disorder, dysthymia) did not moderate the relation between ADHD and suicide attempts among males. No interactions between psychiatric disorders heightened the risk for attempted suicide. Even when including an additional psychiatric disorder (alcohol use disorder), the interaction was not significant
			–	Temporally secondary mood disorders	Kessler et al. (2014)	Secondary mood disorders temporally mediate the relation between ADHD and suicidality. Indirect effects of ADHD through temporally mood disorders substance disorders are statistically significant in predicting suicide ideation (46.0% and 46.5%) and suicide plans (44.2% and 59.1%) among males and females, respectively
			Bipolar disorder (group comp.)	–	Lan et al. (2015)	Bipolar disorder may moderate the relation between ADHD and suicidality. Patients with bipolar disorder and ADHD had a higher risk of attempted suicide than did patients with bipolar disorder only (3.0% vs. 1.1%, *p* = 0.005)
			–	Depression	Lin et al. (2024)	Depression partially mediated the relation between ADHD and risk of thoughts/attempts of suicide. The indirect effect was significant (*ab* = 0.21, 95% CI [0.11, 0.32], *p* < .001)
			Major depressive disorder (interaction term)	–	Ruchkin et al. (2017)	Major depressive disorder did not moderate the relation between ADHD and suicidal ideation (OR = 4.46, 95% CI [0.54, 36.65]) and suicide attempts (OR = 0.342, 95% CI [0.37, 3.14]) in an all-male sample
			Mood disorders (group comp.)	–	Vuijk et al. (2019)	Mood disorder may moderate the relation between ADHD and suicidal thoughts and behaviors. After controlling for comorbid conditions, ADHD was no longer significantly associated with STBs (OR = 1.00, 95% CI [0.67, 1.48], *p* = 0.99), but mood disorder still was (OR = 3.83, 95% CI [2.48, 5.94], *p* < .001). However, odds of STBs in participants with ADHD and mood disorders were significant (*p* < 0.01)
			Major depressive disorder (group comp.)	–	Zahid et al. (2020)	MDD may moderated the relation between ADHD and suicidal behaviors. The relation between ADHD and suicidal behaviors in adolescents with MDD was significant (OR = 1.04, 95% CI [1.01, 1.08], *p* = 0.011) after controlling for demographic factors and psychiatric comorbidities
			–	Anxiety/depression	Chen et al. (2019)	Anxiety/depression partially mediated the relation between ADHD and suicide-related outcomes (i.e., suicidal ideation, suicide plan). The indirect effects of anxiety/depression were statistically significant for suicidal ideation (*β* = 0.22, *p* < .001, proportion explained: 18.76%) and suicide plan (*β* = 0.24, *p* < .001, proportion explained 21.97%). Anxiety/depression fully mediated the relation between ADHD and suicide attempts due to the small sample size for suicide attempts affecting direct effects. The indirect effect of anxiety/depression (*β* = 0.24, *p* < .001, proportion explained: 22.15%) was statistically significant
			–	Depression and anxiety symptoms	Katzenmajer-Pump et al. (2022)	Depressive and anxiety symptoms fully mediated the relation between ADHD and suicidal thoughts and planning. Only the indirect effect was significant, such that both ADHD and anxiety were indirectly related to suicidal thoughts and planning through depression
			–	Depressive and anxiety symptoms	Zhong et al. (2021)	Depressive and anxiety symptoms partially mediated the relation between ADHD symptoms (executive dysfunction, hyperactivity) and suicidal ideation. Under the mediating role of anxiety/depression, executive dysfunction (B = 0.026, β = 0.011, *p* < 0.05) and hyperactivity (B = 0.035, β = 0.015, *p* < 0.05) had significant indirect effects on the risk of SI. For inattention, the direct and indirect effects were not significant
	Anxiety disorders		–	Temporally secondary anxiety disorders	Kessler et al. (2014)	Secondary anxiety disorders temporally mediate the relation between ADHD and suicidal ideation and plans in females (12.2% and 11.3%, respectively), but no significant indirect effect was found in males
			Anxiety disorder (interaction term)	–	Ruchkin et al. (2017)	Anxiety disorder did not moderate the relation between ADHD and suicidal ideation (OR = 0.80, 95% CI [0.09, 6.12]) and suicide attempts (OR = 0.73, 95% CI [0.07, 7.62]) in an all-male sample
			Anxiety disorder (group comp.)	–	Vuijk et al. (2019)	Anxiety disorder may moderate the relation between ADHD and suicidal thoughts and behaviors. After controlling for comorbid conditions, ADHD was no longer significantly associated with STBs (OR = 1.00, 95% CI [0.67, 1.48], *p* = 0.99), but anxiety disorder still was (OR = 1.65, 95% CI [1.11, 2.44], *p* = .01). However, odds of STBs in participants with ADHD and anxiety disorders were significant (*p* < 0.05)
	Disruptive behavior disorders		–	Conduct problems	Chen et al. (2019)	Conduct problems partially mediated the relation between ADHD and suicide-related outcomes (i.e., suicidal ideation, suicide plan, suicide attempts). The indirect effects of conduct problems were statistically significant for suicidal ideation (*β* = 0.09, *p* < .001, proportion explained: 7.35%) and suicide plan (*β* = 0.08, *p* < .001, 7.47%), but not significant for suicide attempts (*β* = 0.10, 8.92%)
			–	Temporally secondary disruptive behavior disorders	Kessler et al. (2014)	Secondary disruptive behavior disorders temporally mediate the relation between ADHD and suicidality (suicide ideation, plans) in the total sample. Indirect effects of ADHD through temporally disruptive behavior disorders are statistically significant in predicting suicide ideation (20.8%) and suicide plans (23.7%) in the total sample
			Conduct disorder (interaction term)	–	Ruchkin et al. (2017)	Conduct disorder did not moderate the relation between ADHD and suicidal ideation (OR = 2.11, 95% CI [0.19, 23.67]) and suicide attempts (OR = 1.23, 95% CI [0.10, 14.70]) in an all-male sample
			Conduct disorder (group comp.)	–	Vuijk et al. (2019)	Conduct disorder may moderate the relation between ADHD and suicidal thoughts and behaviors. After controlling for comorbid conditions, ADHD was no longer significantly associated with STBs (OR = 1.00, 95% CI [0.67, 1.48], *p* = 0.99), but conduct disorder still was (OR = 2.82, 95% CI [1.82, 4.38], *p* < .001). However, odds of STBs in participants with ADHD and conduct disorders were significant (*p* < 0.001)
	ADHD-related factors		ADHD subtype (group comp.)	–	Hinshaw et al. (2012)	ADHD subtype may moderate the relation between ADHD and suicide attempts in an all-female sample. Those with a childhood diagnosis of ADHD-C (22.4%) had significantly higher reports of suicide attempts than those with ADHD-I (7.7%) or comparisons (6%). Significant differences were observed between the comparison group and the ADHD-C group (*d* = 4.5, *p* < .05) and between the ADHD-I and ADHD-C groups (*d* = 3.5, *p* < .05). There were no significant differences between the comparison group and the ADHD-I group (*d* = 1.3)
			Medication use (interaction term)	–	Shoval et al. (2021)	ADHD medication use moderated the relation between externalizing symptoms and suicidality (combined suicidal ideation and attempt). ADHD medication use was associated with less suicidality in children with more externalizing symptoms (significant symptom-by-medication interaction, B = − 0.250; SE = 0.086; *p* = .004), such that for children who were not receiving ADHD medications, there was an association between more externalizing symptoms and suicidality (for a change of 1 SD in symptoms, OR = 1.42; 95% CI [1.33, 1.52], *p* < .001)
			ADHD status (group comp.)	–	Swanson et al. (2014)	ADHD subtypes may moderate the relation between ADHD and suicidality in an all-female sample. The ADHD-C subtype was more strongly associated with suicidality compared to both the ADHD-I subtype and comparison group. Significant differences were observed between the comparison group and the ADHD-C group (*d* = 4.5, *p* < .01) and between the ADHD-I and ADHD-C groups (*d* = 3.5, *p* < .05). There were no significant differences between the comparison group and the ADHD-I group (*d* = 1.3)
			ADHD diagnostic persistence (group comp.)	–	Swanson et al. (2014)	ADHD diagnostic persistence may not moderate the relation between ADHD and suicide attempts in an all-female sample. Although the persistent ADHD group had significantly higher reports of suicide attempts relative to the lifetime comparison group (*d* = 6.7, *p* < .01), the persistent ADHD group did not significantly differ from the transient ADHD group (d = 2.0), and the transient group did not significantly differ from the lifetime comparison group (*d* = 3.4)
			–	Inhibitory control	Swanson et al. (2014)	Inhibitory control did not mediate the relation between ADHD and suicide attempts in an all-female sample. The Indirect effect was not significant
			–	Impulsivity/ behavioral impulsivity	Swanson et al. (2014)	Impulsivity did not mediate the relation between ADHD and suicide attempts in an all-female sample. Indirect effects of impulsivity (measured via two measures) were not significant
			Emotion dysregulation (interaction term)	–	Thompson et al. (2024)	Emotion dysregulation moderated the relation between impulsivity and suicidality (suicidal ideation, suicide attempts). The impulsivity and emotion dysregulation interaction was significant for suicidal ideation and suicide attempts (all *bs* > 0.29, all *ps* < .003)
	Alcohol use and substance use disorders		Alcohol use disorder (interaction term)	–	Kelly et al. (2004)	Alcohol use disorder did not moderate the relation between ADHD and suicide attempts among males. No interactions between psychiatric disorders heightened the risk for attempted suicide. Even when including an additional psychiatric disorder (mood disorder), the interaction was not significant
			–	Temporally secondary substance abuse disorders	Kessler et al. (2014)	Secondary substance abuse disorders temporally mediate the relation between ADHD and suicidality in males. Indirect effects of ADHD through temporally secondary substance disorders are statistically significant among males in predicting suicide ideation and plans (19.8% and 23.5%, respectively). The Indirect effect was only significant among females for suicide ideation (14.6%)
			Alcohol dependence (interaction term)	–	Ruchkin et al. (2017)	Alcohol dependence did not moderate the relation between ADHD and suicidal ideation (OR = 1.33, 95% CI [0.21, 8.53]) in an all-male sample Alcohol dependence moderated the relation between ADHD and suicide attempts (OR = 9.61, 95% CI [1.58, 58.27], *p* < 0.05) in an all-male sample
			Drug dependence (interaction term)	–	Ruchkin et al. (2017)	Drug dependence moderated the relation between ADHD and suicidal ideation (OR = 10.61, 95% CI [1.39, 80.73], *p* < 0.05) in an all-male sample Drug dependence did not moderate the relation between ADHD and suicide attempts (OR = 0.36, 95% CI [0.05, 2.71]) in an all-male sample
	Other psychological and behavioral symptoms		–	Internalizing symptoms	Swanson et al. (2014)	Internalizing symptoms partially mediated the relation between ADHD and suicide attempts in an all-female sample. The indirect effect was significant (IE = 0.11, SE = 0.05, 95% CI [0.03, 0.25])
			–	Externalizing symptoms	Swanson et al. (2014)	Externalizing symptoms did not mediate the relation between ADHD and suicide attempts in an all-female sample. The indirect effect was not significant
			Mania (interaction term)	–	Ruchkin et al. (2017)	Mania did not moderate the relation between ADHD and suicidal ideation (OR = 0.18, 95% CI [0.02, 2.27]) and suicide attempts (OR = 17.67, 95% CI [0.91, 341.60]) in an all-male sample
	Autism spectrum disorder		Autism spectrum disorder (group comp.)	–	Vuijk et al. (2019)	Autism spectrum disorder may moderate the relation between ADHD and suicidal thoughts and behaviors. After controlling for comorbid conditions, ADHD (OR = 1.00, 95% CI [0.67, 1.48], *p* = 0.99) and ASD (OR = 1.13, 95% CI [0.70, 1.82], *p* = 0.63) were no longer significantly associated with STBs. However, odds of STBs in participants with ADHD and ASD were significant (*p* < 0.05)
	Posttraumatic stress disorder		Posttraumatic stress disorder (interaction term)	–	Ruchkin et al. (2017)	PTSD did not moderate the relation between ADHD and suicidal ideation (OR = 0.09, 95% CI [0.01, 1.78]) and suicide attempts (OR = 0.78, 95% CI [0.09, 6.45]) in an all-male sample
Demographics	Sex and gender	13 total (Including: 12 Sex and gender; 1 Age)	Sex (group comp.)	–	Campbell et al. (2019)	Sex may not moderate the relation between ADHD and suicidality. Previous ADHD diagnosis was not associated with an elevated risk of suicide attempts in males (β = − 0.319, *p* = 0.573) or in females (β = − 0.236, *p* = 0.720)
			Gender (interaction term)	–	Chen et al. (2019)	Gender did not moderate the relation between ADHD and suicidality. No interactions between ADHD diagnosis and gender on suicide-related outcomes (i.e., suicidal ideation, suicide plan, suicide attempt) were found in fully adjusted models
			Sex (group comp.)	–	Chen et al. (2020)	Sex may moderate the relation between ADHD and suicidality. Suicidal ideation and attempts in the past 12 months were significantly higher in females with ADHD (OR = 3.81, 95% CI [1.89, 7.68]), but no significant association was found for males with ADHD (OR = 1.00)
			Sex (group comp.)	–	Chronis-Tuscano et al. (2010)	Sex may moderate the relation between ADHD and suicide attempts. Females with ADHD were at greater risk for suicide attempts than males with ADHD (χ^2^ = 3.89, *p* < .05; hazard ratio, 2.57) Sex may not moderate the relation between ADHD and suicidal ideation. Females with ADHD were not at greater risk for later concrete suicidal ideation than males with ADHD (*p* = .17)
			Sex (group comp.)	–	Daviss and Diler (2014)	Sex may moderate the relation between ADHD symptoms and suicidal ideation and behaviors. Variables related to severity of hyperactive/impulsive symptoms were significantly associated with lifetime SB in females only. Specifically, the combined subtype of ADHD was significantly higher for females with lifetime SB (56.3%) than without lifetime SB (23.8%),* p* = .044; CPT-II errors of commission were higher for females with lifetime SB (53.2 ± 9.5) vs. without lifetime SB (45.2 ± 12.6), *p* = .051; and reaction times on the CPT-II were faster for females with lifetime SB (43.2 ± 11.0) vs. without lifetime SB (53.2 ± 11.5), *p* = .015), but these associations were not significant for males
			Sex (group comp.)	–	Forte et al. (2020)	Sex may moderate the relation between ADHD symptoms in childhood and suicidal ideation in adolescence Females in the low, moderate, and high ADHD symptom trajectories were at similar risk of suicidal ideation, even after covariates were added (moderate, OR = 1.0, 95% CI [0.5, 1.7]; high, OR = 1.8, 95% CI [0.7, 5.0]). However, males in the moderate and high ADHD symptom trajectories had a higher risk of suicidal ideation than males in the low trajectory, even after covariates were added (moderate, OR = 4.2, 95% CI [1.2, 14.8]; high, OR 3.6, 95% CI [0.8, 15.1]) Sex may moderate the relation between ADHD symptoms in childhood and suicide attempts in adolescence. The risk of suicide attempts was not higher for females on the high ADHD symptoms trajectory compared to females on the low trajectory after covariates were added (moderate, OR = 1.6, 95% CI [0.8, 3.0]; high, OR = 2.3, 95% CI [0.9, 5.8]). However, males showed an increased risk of suicide attempt on a high ADHD symptom trajectory compared to males on the low trajectory after covariates were added (high, OR = 4.5, 95% CI [1.1, 17.9])
			Sex (interaction term & group comp.)		Galera et al. (2021)	Sex may moderate the relation between specific ADHD symptom profiles in childhood and suicidal ideation and attempt in adolescence. Sex and profile interactions were tested, but results were not reported Secondary analyses via group comparisons found that two profiles with specific combinations of ADHD symptoms and irritability (moderately high ADHD & low irritability profile and low ADHD & moderately high irritability profile) were associated with suicidality in males only. Specifically, the moderately high ADHD & low irritability profile in boys had an odds ratio of 3.35 (95% CI [2.11, 5.33]; effect size = 0.67), and the low ADHD & moderately high irritability profile in boys had an odds ratio of 1.80 (95% CI [1.01, 3.20]; effect size = 0.32). The high ADHD & high irritability profile, however, was associated with suicidality in both females and males. For girls, the odds ratio was 2.39 (95% CI [1.40, 4.10]; effect size = 0.48), and for boys, it was 2.42 (95% CI [1.38, 4.24]; effect size = 0.49). This suggests a more consistent pattern across genders for this specific ADHD profile
			Gender (group comp.)	–	Kelly et al. (2004)	Sex may moderate the relation between ADHD diagnosis and suicide attempts. ADHD was significantly associated with an elevated risk of suicide attempts in males (OR = 2.8, 95% CI [1.28, 6.25]), but not in females (OR = 0.9, 95% CI [0.45, 2.48])
			Sex (group comp.)	–	Kessler et al. (2014)	Sex may moderate the relation between lifetime ADHD diagnosis and lifetime history of suicide attempts. The ORs for ADHD predicting suicide attempts were significantly higher among males (12.3) than females (2.4; χ_1_^2^ = 3.9, *p* = 0.049) Sex may not moderate the relation between lifetime ADHD diagnosis and lifetime history of suicide ideation and plans. The ORs for ADHD predicting suicide ideation and plans are 3.1 and 4.2, respectively and are equivalent for males and females (χ_1_^2^ = 0.6–1.9, *p* = 0.17–0.42)
			Gender (interaction term)	–	Kim et al. (2015)	Gender moderated the relation between high OE (inattention) and suicidal ideation. Specifically, in both the visual sustained and divided attention tasks, the interactions between gender and high OE were statistically significant (*p* < 0.001 and *p* = 0.013, respectively), with significant associations found between high OE and severe suicidal ideation in females Gender did not moderate the relation between high CE (impulsivity in attention tasks) and suicidal ideation in both the visual sustained and divided attention tasks. The interactions between gender and high CE were not significant (*p* = 0.417 and *p* = 0.251, respectively) Gender did not moderate the relation between high RT and suicidal ideation in both the visual sustained and divided attention tasks. The interactions between gender and high RT were not significant (*p* = 0.763 and *p* = 0.425, respectively). However, the interaction between gender and high SD of RT was significant for the visual sustained attention tasks (*p* = 0.002), but not significant for the divided attention tasks (*p* = 0.932)
			Gender (interaction term)	–	Lin et al. (2024)	Gender moderated the relation between ADHD diagnosis and suicidal thought or attempt. The influence of ADHD on thoughts or attempts of suicide (adjusted OR = 0.22, 95% CI [0.06, 0.83]) was significantly more pronounced in males than in females
			Gender (group comp.)	–	Plattner et al. (2007)	Gender may moderate the relation between ADHD combined type and suicidality. Among male delinquent juveniles only, a higher prevalence of the ADHD combined type was found in the currently suicidal group compared to the currently nonsuicidal group (χ^2^ = 8.1, *p* = 0.004). In contrast, among female delinquent juveniles, no significant differences for ADHD combined type were observed between the currently suicidal and nonsuicidal groups (χ^2^ = 3.1, *p* = 0.140). No statistical differences in groups for either gender was found for the ADHD inattentive type or ADHD hyperactive type Gender may moderate the relation between attention problems and suicidality. Among male delinquent juveniles only, the currently suicidal group endorsed significantly more attention problems than the currently nonsuicidal group (F = 13.21, *p* < .001). However, among female delinquent juveniles, no significant differences in attention problems were found between the currently suicidal and nonsuicidal groups (F = 2.96, *p* = .091)
	Age		Age (group comp.)	–	Ben-Yehuda et al. (2012)	Age may moderate the relation between ADHD diagnosis and suicidality (suicide attempt, suicidal ideation). ADHD diagnosis was a more prominent factor in children aged 12 years and below who exhibited suicidal behavior (25.6%), compared to adolescents older than 12 who exhibited suicidal behavior (5.7%)
Peer and social	Social preference	2 total (Including: 2 Bullying; 1 Social preference)	–	Social preference	Meza et al. (2016)	Social preference partially mediated the relation between commission errors (response inhibition) and suicide-related outcomes (i.e., suicidal ideation, suicide attempts) in an all-female sample. The indirect effect was significant for suicidal ideation (*IE* = .0042, *SE* = .0030, 95% CI [.0002, 0122]). The indirect effect was also significant for suicide attempt (*IE* = .0775, *SE* = .0537, 95% CI [.0049, .2257]). Social preference remained a significant partial mediator when peer victimization was entered into the models
	Bullying		–	Bullying victimization	Lin et al. (2024)	Bullying victimization partially mediated the relation between ADHD and risk of thoughts/attempts of suicide. The indirect effect was significant (*ab* = 0.17, 95% CI [0.08, 0.26], *p* < .001)
			–	Peer victimization	Meza et al. (2016)	Peer victimization did not mediate the relation between commission errors (response inhibition) and suicide-related outcomes (i.e., suicidal ideation, suicide attempts) in an all-female sample
Family	Parental stress	2 total (Including: 1 Parental stress; 1 Family function)	–	Parental stress	Gordon and Hinshaw (2017)	Due to the absence of significant correlations between parental stress (parental distress and stress due to dysfunctional interactions in the mother-daughter relationship) and the count of suicide attempts, mediational analyses were not conducted in an all-female sample
	Family function		–	Family function	Chen et al. (2019)	In models that included anxiety/depression, family function partially mediated the relation between ADHD and suicide-related outcomes (i.e., suicidal ideation, suicide plan). The indirect effects of family function were statistically significant for suicidal ideation (*β* = 0.08, *p* < .001, proportion explained: 7.09%) and suicide plan (*β* = 0.18, *p* < .001, 16.76%). Total indirect effects were significant for suicidal ideation (*β* = 0.30, *p* < 0.001, proportion explained: 25.86%) and suicide plan (*β* = 0.42, *p* < .001, 38.73%). Family function fully mediated the relation between ADHD and suicide attempts due to the small sample size for suicide attempts affecting direct effects. The indirect effect of family function (*β* = 0.18, *p* < .001, proportion explained: 16.00%) was statistically significant In models that included conduct problems, family function partially mediated the relation between ADHD and suicide-related outcomes (i.e., suicidal ideation, suicide plan, suicide attempts). The indirect effects of family function were statistically significant for suicidal ideation (*β* = 0.10, *p* < .001, proportion explained: 8.57%), suicide plan, and suicide attempts. Total indirect effects were significant for suicidal ideation (*β* = 0.19, *p* < .001, proportion explained: 15.92%), suicide plan (*β* = 0.27, *p* < .001, 25.26%), and suicide attempt (*β* = 0.29, *p* < .001, 25.88%)
Subjective well-being	Quality of life	1 total	–	Quality of life	Balazs et al. (2018)	Quality of life did not mediate the relation between attention-deficit/hyperactivity problems and suicidal risk. The Indirect effect was not significant (a_3_b_5_ = 0.155, 95% BCa CI [− 0.598, 0.855])
Trauma and adversity	Trauma and adversity	1 total (Including: 1 Trauma and adversity)	Maltreatment (group differences)	–	Guendelman et al. (2016)	Maltreatment may moderate the relation between ADHD diagnosis and suicide attempts in an all-female sample. Females with a childhood diagnosis of ADHD and who were maltreated reported significantly greater suicide attempts than adolescents with ADHD who were not maltreated (χ^2^ = 6.59, *OR* = 1.85, 95% CI [1.14, 3.00]; *p* = .01)

Some articles are listed more than once in the tables below because their findings fall into multiple categories (i.e., the study examines both demographic and psychological factors)

*ADHD* attention-deficit/hyperactivity disorder, *CE* commission error (impulsivity in attention tasks), *CPT-II* continuous Performance Test-II, *group comp* group comparison, *MD* major depression, *MDD* major depressive disorder, *NS* non-signification statistically, *OE* omission error (inattention), *PTSD* posttraumatic stress disorder, *RT* reaction time to correct responses, *SB* suicidal behavior, *Sig*. significant, *STBs* suicidal thoughts and behaviors

### Psychological Factors

#### Affective Disorders

Eleven studies explored the effects of different aspects of affective disorders on the association between ADHD and suicidality. All six studies that included potential mediators found significant evidence of significant mediation. However, the five moderation studies yielded mixed findings. For example, in an all-male sample, the interactions between mood disorders and ADHD on suicide attempts, as well as major depressive disorder and ADHD on suicidal ideation and attempts, were not significant (Kelly et al., 2004; Ruchkin et al., 2017). However, mood disorders and major depressive disorder moderated the relation between ADHD and suicidal behaviors (Vuijk et al., 2019; Zahid et al., 2020). Further, Lan and colleagues (2015) found that patients with bipolar disorder and ADHD had a higher incidence of suicide attempts than did patients with bipolar disorder only.

Three studies found that depression significantly mediated the relation between ADHD and suicidality. For ADHD diagnosis, secondary mood disorders temporally mediated the relation between ADHD and both suicidal ideation and suicide plans (Kessler et al., 2014), while depression partially mediated the link between ADHD and suicidal ideation and attempts (Lin et al., 2024). The relation between conduct problems of ADHD symptoms and suicidal ideation in an all-female sample was partially mediated by depressive symptoms (Cho et al., 2008). Depressive symptoms fully mediated the relations between cognitive and hyperactive problems of ADHD symptoms and suicidal ideation.

Three studies investigated the combined role of depressive and anxiety symptoms as a potential mediator of the association between ADHD and suicidality, which yielded significant results. Depression and anxiety partially mediated the relation between ADHD and suicidal ideation and suicide plan and fully mediated the relation between ADHD and suicide attempts (Chen et al., 2019). In another study, the relation between ADHD and suicidal thoughts and planning was fully mediated by depression and anxiety (Katzenmajer-Pump et al., 2022). For ADHD symptoms, depressive and anxiety symptoms partially mediated the relation between executive dysfunction and suicidal ideation, as well as hyperactivity and suicidal ideation (Zhong et al., 2021). For inattention, the direct and indirect effects were not significant.

#### Anxiety Disorders

Three studies explored anxiety disorders as a mediator or moderator in the ADHD-suicidality link. Vuijk and colleagues (2019) found that having an anxiety disorder moderated the relation between ADHD and suicidal thoughts and behaviors. For female adolescents, anxiety disorders temporally mediated the relation between ADHD and suicidal ideation and plans (Kessler et al., 2014). However, anxiety disorders were not found to be a significant mediator or moderator for male adolescents (Kessler et al., 2014; Ruchkin et al., 2017).

#### Disruptive Behavior Disorders

Four studies examined the impact of conduct disorders on the ADHD-suicidality linkage. Two studies identified disruptive behavior disorders as a significant mediator, while one found it to be a significant moderator. Both conduct problems and temporally secondary disruptive behavior disorders partially mediated the relation between ADHD and suicidal ideation and suicide plans (Chen et al., 2019; Kessler et al., 2014). However, conduct problems were not a significant mediator for suicide attempts (Chen et al., 2019). Moreover, Vuijk and colleagues (2019) found that conduct disorder acted as a moderator between ADHD and suicidality, leading to a significant rise in suicidal thoughts and behaviors. The interaction between conduct disorder and ADHD on suicidal ideation and suicide attempts in a male sample was not significant (Ruchkin et al., 2017).

#### ADHD-related Factors

Four studies explored the moderating and mediating roles of specific ADHD-related factors, finding mixed results based on the construct. In an all-female sample, ADHD subtype moderated the relation between ADHD and suicide attempts, with the combined type showing a stronger association than the inattentive type and comparison group (Hinshaw et al., 2012; Swanson et al., 2014). No significant difference was found between the inattentive type and comparison groups. Emotion dysregulation moderated the relation between hyperactive-impulsive symptoms of ADHD and suicidal behaviors, such that adolescents treated for major depression with high emotion dysregulation and impulsivity were more at risk for suicidal ideation and attempts (Thompson et al., 2024). ADHD medication moderated the relation between externalizing symptoms (including ADHD, ODD, and CD symptoms) and suicidality, such that adolescents with higher levels of externalizing symptoms who were not on medication reported more suicidality, whereas those on medication did not (Shoval et al., 2021). However, ADHD diagnostic persistence was not a significant moderator when comparing persistent ADHD, transient ADHD, and lifetime comparison groups on suicide attempts (Swanson et al., 2014). Additionally, impulsivity and response inhibition, factors related to ADHD’s core symptoms, were not significant mediators of ADHD diagnosis and suicide attempts (Swanson et al., 2014).

#### Alcohol Use and Substance Use Disorders

Two studies explored alcohol- and substance-related moderators in the ADHD-suicidality relation, while one examined the mediating role of secondary substance use disorders. Specifically, Ruchkin and colleagues (2017) found that the interaction between ADHD and alcohol dependence predicted suicide attempts, while the interaction between ADHD and drug dependence predicted suicidal ideation in male juvenile delinquents. Kessler and others (2014) found significant indirect effects of ADHD through substance abuse on suicidal ideation and plans in boys, but only on ideation in girls. In contrast, Kelly and colleagues (2004) observed that the interaction between ADHD and alcohol use disorder did not significantly affect suicide attempts in a male sample.

#### Other Psychological and Behavioral Symptoms

Two studies examined psychological and behavioral symptoms as potential mediators and moderators between ADHD and suicidality. Swanson and colleagues (2014) found that internalizing symptoms partially mediated the relation between ADHD and suicide attempts, while externalizing symptoms did not reach statistical significance. The interaction between ADHD and mania was not significant for suicidal ideation and attempts in juvenile delinquent males (Ruchkin et al., 2017).

#### Autism Spectrum Disorder

One study examined autism spectrum disorder (ASD) as a moderator between ADHD and suicidality. Vuijk and colleagues (2019) found that autism spectrum disorder moderated the relation between ADHD and suicidality, such that the odds of suicidal thoughts and behaviors in adolescents with ADHD and ASD were significantly greater when compared to ADHD alone.

#### Posttraumatic Stress Disorder

One study examined posttraumatic stress disorder (PTSD) as moderator between ADHD and suicidality. Ruchkin and colleagues (2017) found that the co-occurrence of ADHD and PTSD did not increase the risk of suicidal ideation or attempts among juvenile delinquent males.

### Demographic Factors

#### Sex and Gender

Several studies provided information about the impact of biological sex or gender on the relation between ADHD and suicidality, yielding mixed results. Six studies suggested that ADHD in males may be related to a higher risk of suicidality (Forte et al., 2020; Galera et al., 2021; Kelly et al., 2004; Kessler et al., 2014; Lin et al., 2024; Plattner et al., 2007). Three studies found that ADHD diagnosis elevates the risk of suicide attempts more in males relative to females (Kelly et al., 2004; Kessler et al., 2014; Lin et al., 2024). Additionally, gender moderated the relation between ADHD diagnosis and suicidal thoughts, such that the influence of ADHD on suicidal thoughts was significantly more pronounced in males than in females (Lin et al., 2024). Three studies examining the relation between ADHD symptom manifestations and suicidality identified sex/gender differences, highlighting greater variability among males (Forte et al., 2020; Galera et al., 2021; Plattner et al., 2007). Suicidality risk remained significant for males with moderate and/or high ADHD symptom trajectories compared to males on the low trajectory, while females in the low, moderate, and high ADHD symptom trajectories were at similar risk for suicidal ideation and attempts (Forte et al., 2020). Adolescents with high ADHD and irritability were at increased risk for suicidal ideation and attempts regardless of sex/gender (Galera et al., 2021). Among males only, suicidality was also linked to a moderately high ADHD and low irritability profile and a low ADHD with moderately high irritability profile, indicating that even moderate ADHD or irritability levels can influence suicidality in males. Both the ADHD combined type and attention problems were significantly more prevalent in male suicidal groups than male nonsuicidal groups, while female groups were not statistically different (Plattner et al., 2007).

Four studies suggested that females with ADHD may have an elevated risk of suicidality compared to males (Chen et al., 2020; Chronis-Tuscano et al., 2010; Daviss & Diler, 2014; Kim et al., 2015). Two studies found that ADHD diagnosis significantly elevated the risk of suicidal ideation and/or attempts in females with ADHD relative to males with ADHD (Chen et al., 2020; Chronis-Tuscano et al., 2010). Variables related to severity of hyperactive/impulsive symptoms (e.g., combined subtype of ADHD, higher Continuous Performance Test-II (CPT-II) errors of commission, higher CPT-II reaction times) were significantly associated with more lifetime suicidal ideation and behaviors in females, while associations were not significant for males (Daviss & Diler, 2014). Additionally, gender moderated the relation between high omission errors (inattention) and suicidal ideation, such that significant associations between high omission errors and severe suicidal ideation were found in females (Kim et al., 2015).

In contrast, five studies did not find significant sex/gender effects on the ADHD-suicidality relation (Campbell et al., 2019; Chen et al., 2019; Chronis-Tuscano et al., 2010; Kessler et al., 2014; Kim et al., 2015). No interactions between ADHD diagnosis and gender on suicide-related outcomes (i.e., suicidal ideation, suicide plan, suicide attempt) were found in fully adjusted models for sociodemographic variables and mediators (Chen et al., 2019). ADHD similarly increased the risk of suicidal ideation and plans for both males and females, and females with ADHD were not at greater risk for later concrete suicidal ideation than males with ADHD (Chronis-Tuscano et al., 2010; Kessler et al., 2014). A prior ADHD diagnosis was not associated with an elevated risk of suicide attempts in males or females (Campbell et al., 2019). Gender also did not moderate the relation between high commission error (impulsivity in attention tasks) or high RT (reaction time to correct responses) and suicidal ideation (Kim et al., 2015).

#### Age

One study reported information related to age in their results. Ben-Yehuda and colleagues (2012) found that age acted as a moderator, with ADHD diagnosis being a more prominent factor in younger individuals exhibiting suicidality (suicidal ideation or attempts). Specifically, children aged 12 or younger who were suicidal were significantly more likely to have an ADHD diagnosis compared to suicidal adolescents over 12 years old.

### Family Factors

#### Parental Stress

One study examined the effects of parental stress as a mediator between ADHD and suicide attempts; however, no significant correlation was found between parental distress or dysfunctional parent–child interactions and the number of suicide attempts, so mediational analyses were not conducted (Gordon & Hinshaw, 2017).

#### Family Function

One study reported information related to family function in their results. Chen and colleagues (2019) found that family function, as defined by five dimensions of perceived family support, partially mediated the relation between ADHD and suicide-related outcomes (i.e., suicidal ideation, suicide plan) in models that included anxiety/depression. Family function fully mediated the relation between ADHD and suicide attempts due to the small sample size for suicide attempts. In models that included conduct problems, family function also partially mediated the relation between ADHD and suicide-related outcomes (i.e., suicidal ideation, suicide plan, suicide attempts). The indirect effects of family function were significant for all suicide outcomes.

### Peer and Social Factors

#### Social Preference

One study examined the mediating effects of peer social preference (i.e., peer acceptance quantified as the difference between teacher-reported acceptance and rejection ratings of classmates) on the relation between response inhibition and suicidality in an all-female sample (Meza et al., 2016). Self-reports were not utilized to avoid concerns about the accuracy of peer status appraisals. Social preference partially mediated the relation between response inhibition and later suicidal ideation and suicide attempts. Even when accounting for peer victimization, social preference continued to be a significant partial mediator in both models.

#### Bullying

Two studies investigated whether peer victimization (i.e., average of self-reported experiences of being hit, teased to one’s face, and teased behind one’s back) and bullying victimization mediated the relation between ADHD and suicidality (Lin et al., 2024; Meza et al., 2016). Meza and colleagues (2016) found that self-reported peer victimization did not mediate the relation between response inhibition and later suicidal ideation and suicide attempts in an all-female sample. However, bullying victimization in the past 12 months was a significant partial mediator in the relation between ADHD diagnosis and risk of thoughts and attempts of suicide (Lin et al., 2024).

### Subjective Well-Being

One study examined the mediating role of quality of life, measured through a total score encompassing six domains: school, family, peer relations, being alone, somatic health, and mental state (Balazs et al., 2018). Quality of life did not mediate the relation between ADHD problems and suicidal risk. However, while quality of life was not a significant mediator, hyperactivity/impulsivity symptoms were found to be associated with an increase in emotional symptoms and conduct problems. These difficulties were associated with peer relationship challenges and heightened suicide risk.

### Trauma and Adversity Factors

One study explored the role of maltreatment experienced in childhood and/or adolescence and adolescent functioning in females with and without a childhood diagnosis of ADHD (Guendelman et al., 2016). Adolescents with a childhood diagnosis of ADHD who experienced maltreatment reported significantly more suicide attempts than those without maltreatment (i.e., the presence of maltreatment increased the risk of suicide attempts in adolescents with ADHD).

## Discussion

Despite existing research, there remains a relative gap in understanding the nuanced factors influencing suicidality within individuals with significant ADHD symptoms. Adolescence is a critical period for the emergence of suicidality (Cha et al., 2018), yet there is a notable absence of reviews specifically investigating factors that underlie the relation between ADHD and suicidality during this developmental stage. The purpose of this review was to examine if there is a direct association between ADHD and suicidality and to explore what factors moderate or mediate this association. By examining these questions, we address several gaps in the existing literature regarding the relation between ADHD and suicidal behaviors, extend previous research in this area, and offer valuable insights for both researchers and clinicians. These insights can aid in identifying risk factors to prevent crises, developing more effective treatment approaches, and guiding targeted interventions for adolescents with ADHD.

The direct effect of ADHD on suicidality in an adolescent population is a critical finding in this review. Numerous studies demonstrate that adolescents with ADHD are at significantly higher risk of experiencing suicidal ideation and attempts compared to their non-ADHD peers. This increased risk remains substantial even after adjusting for sociodemographic and cognitive factors. Moreover, several studies found that the association between ADHD and suicidality is independent of other psychiatric comorbidities. These findings align with two reviews that have also identified significant direct effects between ADHD and suicidality across the lifespan (Austgulen et al., 2023; Septier et al., 2019). A minority of studies found no direct effect of ADHD on suicidality when accounting for the mediating effects of anxiety and depression as well as other comorbid conditions, highlighting the complex interplay between ADHD and other mental health issues. Nonetheless, the evidence overall supports a direct association between ADHD and suicidality. The impact of ADHD on suicidality may be explained by the UPPS model of impulsivity, which links negative urgency, lack of premeditation, lack of perseverance, and sensation seeking to suicidality (Beach et al., 2022; Whiteside & Lynam, 2001). Additionally, executive dysfunction and emotion dysregulation are associated with both ADHD and suicidality (Bredemeier & Miller, 2015; Groves et al., 2022; Turton et al., 2021).

However, the nature of the relation between ADHD and suicidality is nuanced, with several factors either strengthening or weakening the association, underscoring the importance of considering a broad range of influences when evaluating suicidality in adolescents with ADHD. Overall findings suggest that sex/gender differences may exist, though patterns are inconsistent. Comorbid conditions (e.g., affective disorders, disruptive behavior disorders, anxiety disorders) can also increase the likelihood that an adolescent with ADHD will experience suicidality. In addition, a few studies found significance when considering social preference, bullying victimization, family function, and maltreatment as moderators and mediators. However, the small number of these studies limits the ability to draw concrete conclusions and highlights the need for future research. Substantial heterogeneity across studies presents additional challenges.

### Influences of Psychiatric Comorbidities

Most studies that examined psychiatric comorbidities as mediators found them to have a significant impact on the relation between ADHD and suicidality. All studies that examined affective disorders or combined depression/anxiety symptoms as mediators found significant effects, suggesting that these psychiatric disorders and symptoms help clarify the mechanism through which ADHD may influence suicidality. This is consistent with prior findings which suggest that depression is associated with suicidality and is highly prevalent among individuals with ADHD (Meinzer & Chronis-Tuscano, 2017; Orsolini et al., 2020). Research has identified causal mechanisms contributing to the co-occurrence of ADHD and depression, such as the persistence of ADHD symptoms, reward responsivity, emotion dysregulation, parenting and family factors, and maternal depression (Meinzer & Chronis-Tuscano, 2017).

However, the results regarding affective and anxiety disorders were mixed as to whether the factors were significant moderators and thus additional factors may influence the strength of the ADHD-suicidality relation. Some studies did not find that affective and anxiety disorders were significant moderators in male-only samples, whereas significance was observed in female samples. It is possible that sex/gender differences exist due to differences in the presentation of the disorder, socialization, and hormonal or biological factors (Altemus et al., 2014). To draw clearer conclusions, additional studies are needed to test for interaction effects between ADHD and psychiatric comorbidities, as well as sex/gender differences.

Although fewer studies examined other psychiatric comorbidities, thus limiting the ability to draw concrete conclusions, similar patterns emerged. When considering psychiatric comorbidities (e.g., disruptive behaviors, substance abuse, internalizing symptoms) as mediators in the relation between ADHD and suicidality, significant indirect effects were generally found. In terms of moderators, substance dependence, ADHD subtype in females, emotion dysregulation, ADHD medication, and ASD significantly influenced the strength of the association between ADHD and suicidality. In contrast, alcohol use disorder in males, ADHD persistence, impulsivity, inhibitory control, PTSD, and mania did not significantly moderate the relation between ADHD and suicidality. Findings related to disruptive behavior were inconsistent, with one study showing a significant effect and another finding no significance in an all-male sample. Overall findings suggest that psychiatric comorbidities can influence the ADHD-suicidality relation by strengthening, weakening, or otherwise modifying it through their roles as moderators or mediators. These findings are important because youth with ADHD are likely to have other mental health conditions (Larson et al., 2011). It may be beneficial to address comorbid conditions in individuals with ADHD to reduce the risk of suicidal behaviors; tailoring interventions to specific comorbidities could improve prevention strategies and outcomes for those at higher risk for suicide.

### Influences of Sex/Gender

Most studies examining sex/gender as a potential moderator suggest that significant differences exist in the relation between ADHD and suicidality based on sex/gender; however, the results are mixed regarding which biological sex or gender experiences greater suicidality. Given these inconsistencies, it is challenging to draw clear conclusions about the role of sex/gender in moderating the ADHD-suicidality relation. Ten studies indicated that sex/gender may act as a moderator because the strength and effect of ADHD on suicidality differed between male and female groups. Six of these studies found that ADHD was significantly associated with suicidality in males but not in females, such that ADHD diagnosis, ADHD combined type, moderate and high ADHD symptoms, or attention problems were associated with elevated risks of suicidal ideation and suicide attempts in males relative to females. However, four studies found that ADHD is significantly associated with suicidality in females and not males, such that ADHD diagnosis and variables related to severity of hyperactive/impulsive symptoms were associated with elevated risk of suicidal ideation and suicide attempts in females. Research in youth suicidal behavior suggests higher past year and lifetime prevalence of suicidal ideation and attempts in females, but higher rates of death by suicide in males (Rhodes et al., 2014). A systematic review from Rhodes and colleagues (2014) found that differences in epigenetic mechanisms, neurodevelopment and psychopathology, types of mental disorders, and adaptations to pain may account for these sex/gender differences. In this review, several studies that controlled for sex/gender found that ADHD was significantly associated with suicidality. This suggests that ADHD may be a risk factor for suicidality regardless of sex or gender. The studies that found sex/gender differences may have captured unique developmental, social, or contextual factors that interact with ADHD to influence suicidality, whereas those that did not find differences may have examined broader or more heterogeneous samples that masked these effects.

Additional possible explanations that may contribute to these inconsistent findings include that the relation between ADHD and suicidal outcomes is complex and distinct differences between particular suicidal outcomes (e.g., suicide attempts, suicidal ideation) may exist (Manor et al., 2010; Romanelli et al., 2022). Therefore, when the included studies showed large variability in methodologies and measures used to assess ADHD and suicidality, with 14 different measures capturing ADHD and 18 measures capturing suicidality, it is possible that differences in assessment approaches contributed to inconsistencies in findings. Moreover, the observed group differences of sex/gender could be due to factors such as sample size or statistical power, which may limit the ability to detect true moderation effects. For example, Chronis-Tuscano and colleagues (2010) noted that the small sample size of girls with ADHD was a significant limitation, as a larger sample with greater statistical power could have allowed for the prediction of additional outcomes. Future studies need to make significant efforts to recruit females with ADHD and adolescents with suicidality to allow for statistical analyses that can accurately examine the effects of sex/gender and explore the mechanisms underlying the ADHD-suicidality relation in this developmental period.

### Influences of Additional Psychosocial Factors

It is notable that few studies have examined the mediating role of various psychosocial factors in the relation between ADHD and suicidality. Given the low number of studies exploring these factors, conclusions remain tentative and underscore the need for further research to clarify the relations and underlying mechanisms. Peer relationships take on heightened significance during adolescence, as teens shift their primary attachment from parents to peers (Fuligni & Eccles, 1993). In the current systematic review, peer and social factors included aspects of social preference and bullying. In an all-female sample, social preference partially mediated the relation between response inhibition and suicidal ideation and attempts, while peer victimization did not (Meza et al., 2016). Peer victimization is considered a more direct and overt form of interpersonal problems (e.g., physical aggression, teasing) compared to social preference, which encompasses being isolated or rejected by peers (Meza et al., 2016). However, Lin and colleagues (2024) found that bullying victimization (i.e., exposure to bullying) partially mediated the relation between ADHD and risk of suicidal ideation and attempts. While both males and females may report similar frequencies of social aggression (e.g., being gossiped about), adolescent females tended to ruminate on it more and experience greater distress than adolescent males (Paquette & Underwood, 1999). Chen and colleagues (2020) identified possible pathways from school bullying to suicidality in adolescents with ADHD, with personal competence and psychological well-being either originating or mediating this relation. More studies examining peer and social factors are needed, and sex/gender differences may help explain the findings in these areas. Family function also partially mediated the relation between ADHD and suicide-related outcomes (Chen et al., 2019). Family can play a significant role in the lives of adolescents, influencing their psychological well-being and development (Hazen et al., 2008; Shek, 1998).

These findings may underscore the need to consider broader psychosocial factors, such as family dynamics and peer relationships, when screening and intervening for suicidality in adolescents with ADHD. These findings also support a stress-diathesis model of suicide, in which negative life events can trigger stress that leads to suicidal behaviors (Van Heeringen, 2012). Therefore, effective intervention approaches should address not just ADHD symptoms but also these surrounding contexts, focusing on improving family relationships and social skills to better manage and reduce suicidal risks. For instance, several family based treatments for suicidal ideation and behavior exist that are tailored to adolescents (e.g., CBT + Parent Training, CBT + Systemic Principles, DBT + Family Training, Systemic Principles, and Psychoeducation; Frey et al., 2022). Interventions such as these could be adapted to meet the needs and experiences of adolescents with ADHD.

### Implications

The findings from this systematic review have important implications for both research and clinical practice. General suicide prevention programs for children and adolescents exist, but the evidence supporting their effectiveness is still limited (Witt et al., 2021). Given the robust association between ADHD and suicidality, there is a clear need for targeted interventions that address the unique risks faced by adolescents with ADHD. However, there are no interventions specifically designed to address suicide in individuals with ADHD (Fitzgerald et al., 2019). Targeted interventions should not only address ADHD symptoms directly but also consider the broader psychosocial context, including comorbid psychiatric conditions, family dynamics, and the potential impact of peer relationships. Moreover, the identification of key moderators and mediators highlights the importance of personalized approaches to screening and treatment. Primary care providers may not recognize ADHD as a suicide risk factor, leading to insufficient assessment and intervention for preventing suicidality in adolescents with ADHD (Malik et al., 2023). Additionally, clinicians should be aware of the various factors that may influence the risk of suicidality in adolescents with ADHD and tailor their screenings and interventions accordingly. For example, addressing depressive symptoms, improving family functioning, and fostering psychological resilience may be particularly effective strategies for reducing suicidality in this population. Findings from this study may aid clinicians and educators in implementing effective screening and monitoring practices (e.g., suicide risk assessments) to identify adolescents with ADHD who are at the highest risk of suicidality, facilitating the application of safety planning and these targeted interventions. This ultimately allows for effective suicide prevention to take place and reduces the overall risk of suicidality among adolescents with ADHD.

### Methodological Considerations and Limitations

There are several limitations to consider. As previously mentioned, several key factors were only investigated in one or two included studies, which limits the ability to generalize these findings and draw concrete conclusions. This highlights the need for further research to ensure sufficient data is available for robust meta-analyses in future. In addition, the authors’ decision to include only studies examining moderating or mediating variables in the current study resulted in the exclusion of multiple studies on predictors, potentially limiting the comprehensiveness of the review. Moreover, the search terms used in this review to identify relevant articles related to ADHD did not cover all aspects of the condition, such as symptoms of hyperactivity, impulsivity, and inattention. Instead, given our results, it is probable that the search terms primarily captured studies focusing on ADHD diagnosis or those explicitly measuring ADHD symptoms. Therefore, several studies might have been missed that examined these constructs in relation to other mental health conditions. Another limitation of this systematic review is the inconsistency in the diagnostic criteria and comorbidity inclusion across the studies. For instance, some studies used the ICD-9 criteria, while others relied on DSM-IV or DSM-IV-TR, leading to potential variability in how ADHD and comorbid conditions were defined and measured (American Psychiatric Association [APA], 1994; APA, 2000; World Health Organization [WHO], 1978). Some studies included participants with a broad range of comorbidities, whereas others were more selective, which may have impacted the comparability of findings across studies. Most studies also did not control for or indicate whether their participants were medicated for ADHD. Medication use may serve as an unaddressed confounding or mediating factor within this review. Finally, study selection and data extraction were conducted by a single reviewer. While this approach may increase the risk of selection and extraction bias, clearly defined inclusion and exclusion criteria were intended to minimize such risks. The review also did not include a formal assessment of risk of bias or study quality, which is a methodological limitation because it limits the ability to evaluate the strength and reliability of the findings from the included studies.

### Future Directions

As mentioned, this systematic review found several factors that were only evaluated by one to two studies, which limits the generalizability and robustness of the findings related to these factors. For example, studies concerning family factors, peer and social influences, subjective well-being, trauma and adversity, and ADHD medication were sparse. Even within factors covered by multiple studies, such as demographic variables and psychiatric comorbidities, noticeable gaps remain. No studies we are aware of looked at cultural and ethnic backgrounds or socioeconomic status as moderators, and psychiatric comorbidities such as ASD and personality disorders were scarce. For example, ASD and borderline personality disorder were associated with higher rates of suicidality (May et al., 2021). Given the high comorbidity rates with ADHD, it could be beneficial to explore these factors in the context of ADHD and suicidality (Masi & Gignac, 2015).

There is also a need for research to focus on protective factors that lower the risk of suicidality rather than just identifying risk factors. Beyond a single article on medication use, no articles examined resilience and protective factors. This gap is particularly concerning, as protective factors play a crucial role in mitigating the risk of suicidality. For example, strong social support or high self-esteem can buffer against the negative impacts of stress and psychiatric comorbidities, reducing the likelihood of suicidal thoughts and behaviors (Kleiman & Liu, 2013; Rizwan & Ahmad, 2010). Psychological factors that foster resilience enable individuals to navigate challenges more effectively and maintain psychological well-being despite adversity, which is a significant component of suicide interventions (Johnson et al., 2011). The absence of studies exploring these factors leaves a significant gap in understanding how protective factors might influence the relation between ADHD and suicidality. Addressing these factors in future research could provide valuable insights for developing targeted interventions for this vulnerable population that not only address risk factors but also strengthen the protective mechanisms that safeguard against suicidality.

Finally, all studies incorporated in this review gathered quantitative data. Adopting a mixed-method design could prove beneficial for capturing rich qualitative data regarding the underlying factors influencing suicidality for adolescents with ADHD. As more comprehensive data on the association between ADHD and suicidality is gathered, there is a growing need for future meta-analyses that could offer more accurate risk estimates. Additionally, future reviews should consider incorporating formal risk of bias and quality assessments to their methodology to enhance the rigor of the findings.

## Supplementary Information

Below is the link to the electronic supplementary material.

Supplementary file1 (DOCX 29 KB)

## Data Availability

No datasets were generated or analyzed during the current study.
