# Supplementary material for: ADHD and Suicidality in Adolescents: A Systematic Review of Moderators and Mediators
Source: Clin Child Fam Psychol Rev. 2025 Jun 19;28(3):573–611. doi: 10.1007/s10567-025-00531-9 (PMC12634774; doi:10.1007/s10567-025-00531-9)
Supplement: Supplementary file 1 — Supplementary file1 (DOCX 29 KB) [file 10567_2025_531_MOESM1_ESM.docx]

**Table 1. Study characteristics of all included full-text articles**

| **Study** | **Country** | **Study Design** | **Measure(s) of ADHD** | **Measure(s) of Suicidality** | **Measure(s) of additional constructs** | **Participant characteristics** | | |
| --- | --- | --- | --- | --- | --- | --- | --- | --- |
|  |  |  |  |  |  | **Sample size** | **Gender distribution** | **Age** |
| Balazs et al. (2018) | Hungary | Cross-sectional analytical study  Quantitative study | Hyperactivity/ inattention via the SDQ hyperactivity/ inattention subscale | Suicidal risk (no/low, medium/high suicidal risk) via the M.I.N.I. Kid | Quality of life (total score from domains of school, family peer relations, being alone, somatic health, mental state) via the ILK | n = 134 | 72 (53.7%) males; 62 (46.3%) females | M_age_ = 14.48 (1.34)  Age range = 13 to 18 years |
| Ben-Yehuda et al. (2012) | Israel | Retrospective study  Quantitative study | Previous ADHD diagnosis via a medical chart review (ICD-10) | Suicide attempt or suicidal ideation during a 3-year period via a medical chart review (ICD-10) | Age | n = 266  36 (13.5%) with ADHD; 230 (86.5%) without ADHD | 83 (31.2%) males; 183 (68.8%) females | M_age_ = not reported  39 children (under the age of 12 years); 227 adolescents (ages 12-18 years) |
| Campbell et al. (2019) | Sweden | Longitudinal study  Quantitative study | Previous ADHD diagnosis via medical chart review (ICD-9: 314, 314J, 314W, or 314X; ICD-10: F90.0-9) or if the subject had a prescription for ADHD medication | Suicide attempts via a medical chart review (ICD-10: X60-X84) in CDR | Sex | n = 4,816  1648 (61.3%) males with ADHD; 983 (46.2%) females with ADHD | 2,688 (55.8%) males; 2,128 (44.2%) females | M_age_ = not reported  Age range = 8 to 18 years |
| Chen et al. (2019) | Taiwan | Cross-sectional analytical study  Quantitative study | ADHD diagnosis via the K-SADS-E | Suicidal ideation, suicide plans, and attempts in the past 6 months via the K-SADS-E | Gender  Family function via the Family APGAR   Psychiatric comorbid conditions (conduct problems and anxiety/depression) via the CBCL | n = 4,739  412 (8.7%) with ADHD; 4327 (91.3%) without ADHD | 2,458 (51.9%) males; 2,281 (48.1%) females | M_age_ = 11.11 (1.79) with ADHD; 11.3 (1.77) without ADHD  Age range = 7 to 15 years |
| Chen et al. (2020) | Taiwan | Cross-sectional analytical study  Quantitative study | ADHD diagnosis via ADHD module of the M.I.N.I. Kid | Suicidal ideation and attempts in last 12 months via the K-SADS-E | Sex | n = 203   203 (100%) with ADHD | 160 (78.8%) males; 43 (21.2%) females | M_age_ = 14.0 (1.5)  Age range = 12 to 18 years |
| Cho et al. (2008) | South Korea | Cross-sectional analytical study  Quantitative study | ADHD symptoms via the CASS: Short | Suicidal ideation in the past month via the RSIQ | Depressive symptoms via the CDI | n = 788 | 788 (100%) females | M_age_ = 16.28 (0.95)  Age range = not reported |
| Chronis-Tuscano et al. (2010) | USA | Longitudinal study  Quantitative study | ADHD diagnosis in year 1 based on DSM-IV criteria via the DISC-IV and IRS | Suicide attempts across years 6 through 14 if parent or child reported attempted suicide; DISC-IV in year 9 assessment  Concrete suicidal ideation in the past 6 months if parent or child reported that participant had considered a specific suicidal plan | Sex | n = 248  125 (50.4%) with ADHD; 123 (49.6%) without ADHD | 207 (83.5%) males; 41 (16.5%) females | M_age_ = not reported  Year 1:  Age range = 4 to 6 years  Year 6:  Age range = 9 to 11 years  Year 14:  Age Range = 17 to 18 years |
| Daviss & Diler (2014) | USA | Cross-sectional analytical study  Quantitative study | ADHD diagnosis via the DSM-IV-TR criteria  ADHD symptoms via the K-SADS-PL  Current ADHD symptom severity via the ARS  ADHD severity via the CPT-II | Lifetime suicidal ideations and behaviors via the K-SADS-PL | Sex | n = 101  101 (100%) with ADHD | 64 (63.4%) males; 37 (36.6%) females | M_age_ = 14.6 (2.1) with lifetime SBs, 13.5 (1.8) without lifetime SBs  Age range = 11 to 18 years |
| Forte et al. (2020) | Canada | Longitudinal study  Quantitative study | ADHD symptoms during childhood (from 6 to 12 years) via Behavior Questionnaire | Past-year suicidal ideation and attempt (from 13 to 17 years) via the MIA (“in the past 12 months, did you ever seriously think of attempting suicide”; if answered with “yes,” item “in the past 12 months, how many times did you attempt suicide?”) | Sex | n = 1,407 | 665 (47.26%) males; 742 (52.74%) females | M_age_ = not reported  Age range = 6 to 12 years for ADHD; 13, 15, and 17 years for suicidality |
| Galera et al. (2021) | Canada | Longitudinal study  Quantitative study | ADHD symptoms during childhood (from 6 to 12 years) via Behavior Questionnaire | Suicidal ideation and attempt (from 13 to 17 years) via the MIA | Sex | n = 1,407 | 665 (47.26%) males; 742 (52.74%) females | M_age_ = not reported  Age range = 6 to 12 years for ADHD; 13, 15, and 17 years for suicidality |
| Gordon & Hinshaw (2017) | USA | Longitudinal study  Quantitative study | ADHD diagnosis via the SNAP-IV and DISC-IV | Suicide attempts via the Barkley Suicide Questionnaire & FIP | Parental stress via the PSI – Short Form | Wave 1:  n = 228 140 (61.4%) with ADHD; 88 (38.6%) without ADHD   Wave 2: n = 209  Wave 3: n = 216 | Wave 1:  228 (100%) females  Wave 2:  209 (100%) females  Wave 3:  216 (100%) females | Wave 1: M_age_ = 9.6  Age range = 6 to 12 years  Wave 2: M_age_ = 14.2  Age range = 11 to 18 years  Wave 3:  M_age_ = 19.6  Age range = 17 to 24 years |
| Guendelman et al. (2016) | USA | Longitudinal study  Quantitative study | ADHD diagnosis via the SNAP-IV and DISC-IV | Suicide attempts via the Barkley Suicide Questionnaire | Maltreatment in childhood and/or adolescence via chart review using the BIQ, FIP, Hot Sheet, and Report from the University of California Berkley Summer Program | Wave 1:  n = 140 with ADHD   Wave 2: n = 128 with ADHD  Wave 3: n = 130 with ADHD  Some relevant analyses included 88 comparison girls without ADHD in wave 1 | Wave 1:  140 (100%) females  Wave 2:  128 (100%) females  Wave 3:  130 (100%) females | Wave 1: M_age_ = 9.6 (1.7)  Age range = 6 to 12 years  Wave 2: M_age_ = 14.3  Age range = 11 to 18 years  Wave 3:  M_age_ = 19.7  Age range = 17 to 23 years |
| Hinshaw et al. (2012) | USA | Longitudinal study  Quantitative study | ADHD diagnosis via the SNAP-IV and DISC-IV | Suicide attempts via the Barkley Suicide Questionnaire & FIP | ADHD subtype via DISC-IV | Wave 1:  n = 228 140 (61.4%) with ADHD; 88 (38.6%) without ADHD   Wave 2: n = 209  Wave 3: n = 216 | Wave 1:  228 (100%) females  Wave 2:  209 (100%) females  Wave 3:  216 (100%) females | Wave 1: M_age_ = 9.6  Age range = 6 to 12 years  Wave 2: M_age_ = 14.2  Age range = 11 to 18 years  Wave 3:  M_age_ = 19.6  Age range = 17 to 24 years |
| Katzenmajer-Pump et al. (2022) | Hungary | Cross-sectional analytical study  Quantitative study | ADHD diagnosis via previous ADHD diagnosis by a clinician and the M.I.N.I. Kid | Suicidal thoughts and planning via the M.I.N.I. Kid | Depression symptoms via the M.I.N.I. Kid  Anxiety symptoms via the M.I.N.I. Kid | n = 185  89 (48.1%) with ADHD; 96 (51.9%) without ADHD | 127 (68.6%) males; 58 (31.4%) females | M_age_ = 14.79 (1.48)  Age range = 13 to 18 years |
| Kelly et al. (2004) | USA | Cross-sectional analytical study  Quantitative study | ADHD diagnosis via K-SADS | Suicide attempts via self-report from participants and parents (item asking about attempted suicide and estimated age at time of these events) | Gender  Substance use disorders via the expanded version of the substance use disorders section of the SCID  Non-substance related DSM-defined psychiatric disorders via the K-SADS | n = 503  102 (20.3%) with ADHD; 401 (79.7%) without ADHD | 315 (62.6%) males; 188 (37.4%) females | M_age_ = 16.7 (1.3) for non-attempters; 16.4 (1.4) for suicide attempters  Age range = 12.2 to 19.0 years |
| Kessler et al. (2014) | USA | Cross-sectional analytical study  Quantitative study | Lifetime prevalence of ADHD diagnosis via the DSM-IV/CIDI, K-SADS-PL | Lifetime history of suicidal behaviors (suicide ideation, plans, and attempts) via a modified version of the suicidal behavior module of the CIDI | Sex  Temporally secondary mental disorders (anxiety, mood, disruptive behavior, substance disorders) via the CIDI | n = 6,483  8.1% with lifetime ADHD; 6.3% with 12-month prevalence of ADHD | Not reported | M_age_ = not reported  Age range = 13 to 17 years |
| Kim et al. (2015) | South Korea | Cross-sectional analytical study  Quantitative study | ADHD symptoms (visual sustained attention, divided attention) via computerized CAT | Suicidal ideation via SSI | Gender | n = 2,462 | 1,021 (41.5%) males; 1,441 (58.4%) females | M_age_ = 17.3 (0.6)  Age range = 14 to 19 years |
| Lan et al. (2015) | Taiwan | Retrospective study  Quantitative study | ADHD diagnosis via medical chart review (ICD-9-CM: 314) | Suicide attempts via medical chart review (ICD-9-CM: E950-E959) | Bipolar disorder via medical chart review (ICD-9-CM: 296.0x, 296.1x) | n = 2,000  500 (25%) with ADHD; 1500 (75%) without ADHD | 1,324 (66.2%) males; 676 (33.8%) females | M_age_ = 19.11 (2.84)  Age range = 15 to 24 years |
| Lin et al. (2024) | Australia | Longitudinal study  Quantitative study | ADHD diagnosis by age 10 via parent report | Suicidal thought or attempt over the past 12 months at age 14 via self-report | Gender  Depression at age 12 via SMFQ  Bullying victimization at age 12 via self-report | n = 3,696  133 (3.6%) with ADHD; 3563 (96.4%) without ADHD | 1,899 (51.4%) males; 1,797 (48.6%) females | Age range of relevant variables = 10 to 14 years |
| Meza et al. (2016) | USA | Longitudinal study  Quantitative study | Response inhibition at wave 1 via the CPT | Suicide attempts at wave 3 via the Barkley Suicide Questionnaire | Peer social preference at wave 2 via the DSPS  Peer victimization at wave 2 via the social relationships interview | Wave 1:  n = 228 140 (61.4%) with ADHD; 88 (38.6%) without ADHD  Wave 2: n = 209  Wave 3: n = 216 | Wave 1:  228 (100%) females  Wave 2:  209 (100%) females  Wave 3:  216 (100%) females | Wave 1: M_age_ = 9.6  Age range = 6 to 12 years  Wave 2: M_age_ = 14.2  Age range = 11 to 18 years  Wave 3:  M_age_ = 19.6  Age range = 17 to 24 years |
| Ruchkin et al. (2017) | Russia | Cross-sectional analytical study  Quantitative study | ADHD diagnosis via K-SADS-PL and DSM-IV criteria | Lifetime suicidal ideation and suicide attempts via K-SADS-PL, two items on YSR, and three items on BDI | Psychiatric comorbid conditions (major depressive disorder, mania, anxiety disorder, posttraumatic stress disorder, conduct disorder, alcohol dependence, drug dependence) via K-SADS-PL | n = 370  64 (17.3%) with ADHD; 306 (82.7%) without ADHD | 370 (100%) males | M_age_ = 16.36 (0.84)  Age range = 14 to 19 years |
| Shoval et al. (2021) | USA | Longitudinal study  Quantitative study | ADHD hyperactivity symptoms via the KSADS-5 | Past or current suicidality (suicidal ideation and attempts) via the KSADS-5 | Prescribed medication via the Medication Inventory from the PhenX Instrument | Wave 1: n = 11,878  2550 (25.5%) with ADHD; 9,328 (74.5%) without ADHD  Wave 2:  n = 11,077 | Wave 1: 6196 (52.2%) males; 5682 (47.8%) females | Wave 1: M_age_ = 9.9 (0.6) Age range = 9 to 11 years  Wave 2:  M_age_ = 10.9 (0.6) Age range = 10 to 12 years |
| Swanson et al. (2014) | USA | Longitudinal study  Quantitative study | ADHD diagnosis via the DISC-IV at wave 1 | Suicide attempts via the Barkley Suicide Questionnaire and FIP | Externalizing symptoms via the CBCL and TRF  Internalizing symptoms via the CBCL, TRF, and CDI  Response inhibition via the CPT and CUL  Impulsivity via the SNAP-IV  ADHD type via the SNAP-IV | Wave 1:  n = 228 140 (61.4%) with ADHD; 88 (35.6%) without ADHD   Wave 2:  n = 209  Wave 3:  n = 216 | Wave 1: 228 (100%) females  Wave 2:  209 (100%) females  Wave 3:  216 (100%) females | Wave 1:  M_age_ = 9.1  Age range = 6 to 12 years  Wave 2:  M_age_ = 14.2 Age range = Not reported Wave 3:  M_age_ = 19.6  Age range = Not reported |
| Thompson et al. (2024) | USA | Longitudinal study  Quantitative study | Impulsivity via the hyperactive-impulsive symptoms of ADHD from the M.I.N.I. Kid | Lifetime history of suicidal ideation and suicide attempts via C-SSRS | Emotion dysregulation via the angry/depressed subscale of the CALS | n = 344 | 107 (31%) males; 237 (68.9%) females | M_age_ = not reported  Age range =12 to 15 years at baseline (2 year follow up) |
| Vuijk et al. (2019) | USA | Cross-sectional analytical study  Quantitative study | ADHD diagnosis via  the DSM-IV-TR criteria and 12 youth received a KSADS-E | Suicidal thoughts and behaviors via two items from the CBCL and one item from the CSI-IV | Psychiatric comorbid conditions (anxiety, autism spectrum disorder, conduct disorder, mood disorder) via the CBCL, CSI-IV, and SRS | n = 758  454 (59.9%) with ADHD; 304 (40.1%) without ADHD | 482 (63.6%) males; 276 (36.4%) females | M_age_ = 11.1 (3.2)  Age range = 6 to 17 years |
| Plattner et al. (2007) | Austria | Cross-sectional analytical study  Quantitative study | ADHD diagnosis via the M.I.N.I. Kid | Lifetime and current suicidality via the M.I.N.I. Kid  Suicidal ideation via the MAYSI-2 | Gender | n = 319  125 (39.2%) with ADHD; 194 (60.8%) without ADHD | 266 (83.4%) males; 53 (16.6%) females | M_age_ = 16.67 (1.45)  Age range = 14 to 21 years |
| Zahid et al. (2020) | USA | Cross-sectional analytical study  Quantitative study | Secondary discharge ADHD diagnosis via medical chart review (ICD-9: 314.00 or 314.01; and CCS codes) | Suicidal behaviors via medical chart review (ICD-9 and CCS codes) | Primary discharge major depressive disorder diagnosis via medical chart review (ICD-9: 296.20-296.26 or 296.30-296.36; and CCS codes) | n = 141,530  22,665 (16%) with ADHD; 118,865 (84%) without ADHD | 40,479 (28.6%) males; 101,051 (71.4%) females | M_age_ = 15.3 without ADHD, 15.1 with ADHD  Age range = 12 to 18 years |
| Zhong et al. (2021) | China | Cross-sectional analytical study  Quantitative study | ADHD symptoms via the ASRS-5 | Lifetime suicidal ideation via the BSS | Depressive symptoms over the last 7 days via the QIDS-SR  Anxiety symptoms over the last 2 weeks via GAD-7 | n = 904 | 48.2% males; 51.8% females | M_age_ = 18.2 (0.9)  Age range = 16 to 22 years |

**_Note regarding the measures’ names:_** _ADHD Rating Scale (ARS; DuPaul et al., 1998); Adult ADHD Self-Report Screening Scale for DSM-5 (ASRS-5; Kessler et al., 2005; Ustun et al., 2017); Background Information Questionnaire (BIQ; Hinshaw, 2002); Barkley Suicide Questionnaire (Barkley & Murphy, 2006); Beck Depression Inventory (BDI; Beck et al., 1961); Beck Scale for Suicide Ideation (BSS; Beck et al., 1979); Behavior Questionnaire (Forte et al., 2020); Cancel underline task (CUL; Rourke & Orr, 1977); Cause of Death Registry (CDR); Child Behavioral Checklist (CBCL; Achenbach, 1999); Children’s Depression Inventory (CDI; Kovacs, 1992); Children’s Affective Lability Scale (CALS; Gerson et al., 1996); Child Symptom Inventory, fourth edition (CSI-IV; Gadow & Sprafkin, 2002); Columbia Suicide Severity Rating Scale (C-SSRS; Posner et al., 2008); Composite International Diagnostic Interview (CIDI; Kessler & Üstün, 2004); modified version of the suicidal behavior module of the Composite International Diagnostic Interview (CIDI; Nock et al., 2009); Comprehensive Attention Test (CAT; Yoo et al., 2009); Conners/Wells Adolescent Self-Report Scale (CASS; Conners et al., 1997); Connors’ Continuous Performance Test-II (CPT-II; Conners et al., 2000); Diagnostic and Statistical Manual of Mental Disorders, 4th edition, revised (DSM-IV-TR; American Psychiatric Association, 2000); Diagnostic Interview Schedule for Children, Fourth Edition (DISC-IV; Shaffer et al., 2000); Dishion Social Acceptance Scale (DSPS; Dishion, 1990); Family APGAR (Smilkstein et al., 1982); Family Information Packet (FIP); Generalized Anxiety Disorder Scale-7 (GAD-7; Spitzer et al., 2006); Hot Sheet (Briscoe-Smith & Hinshaw, 2006); Impairment Rating Scale (IRS; Fabiano et al., 2006); International Classification of Diseases, Ninth Revision, Clinical Modification (ICD-9-CM); International Classification of Diseases, 10th edition (ICD-10); Kiddie Schedule for Affective Disorders and Schizophrenia (K-SADS; Kaufman et al., 1997); Massachusetts Youth Screening Instrument (MAYSI-2; Grisso & Barnum, 2000); Mental Health and Social Inadaptation Assessment for Adolescents (MIA; Côté et al., 2017); Mini-International Neuropsychiatric Interview for Children and Adolescents (M.I.N.I. Kid; Sheehan et al., 2010); Parenting Stress Index (PSI; Abidin et al., 2006); Quick Inventory of Depressive Symptomatology-Self Report (QIDS-SR; Rush et al., 2003); Reynolds Suicidal Ideation Questionnaire (RSIQ; Reynolds, 1991); Strengths and Difficulties Questionnaire (SDQ; Goodman, 1997); Scale for Suicide Ideation (SSI; Beck & Steer, 1991); Short Mood and Feelings Questionnaire (SMFQ; Turner et al., 2014); Social Relationships Interview (Meza et al., 2017); Social Responsiveness Scale (SRS; Constantino, 2021); Structured clinical interview for DSM-III-R disorders (SCID; Spitzer et al., 1992); Swanson, Nolan, and Pelham, Version IV (SNAP-IV; Swanson et al., 2001); Teacher Report Form (TRF; Achenbach, 1991); Youth Self-Report (YSR; Achenbach, 1991)_
